# Niche differentiation in a postglacial colonizer, the bank vole *Clethrionomys glareolus*

**DOI:** 10.1002/ece3.7637

**Published:** 2021-05-17

**Authors:** Marco A. Escalante, Michaela Horníková, Silvia Marková, Petr Kotlík

**Affiliations:** ^1^ Laboratory of Molecular Ecology Institute of Animal Physiology and Genetics of the Czech Academy of Sciences Liběchov Czech Republic; ^2^ Department of Zoology Faculty of Science Charles University Prague Czech Republic

**Keywords:** cryptic refugia, ecological niche modeling, intraspecific variation, Last Glacial Maximum, MaxEnt, *Myodesglareolus*

## Abstract

Species‐level environmental niche modeling has been crucial in efforts to understand how species respond to climate variation and change. However, species often exhibit local adaptation and intraspecific niche differences that may be important to consider in predicting responses to climate. Here, we explore whether phylogeographic lineages of the bank vole originating from different glacial refugia (Carpathian, Western, Eastern, and Southern) show niche differentiation, which would suggest a role for local adaptation in biogeography of this widespread Eurasian small mammal. We first model the environmental requirements for the bank vole using species‐wide occurrences (210 filtered records) and then model each lineage separately to examine niche overlap and test for niche differentiation in geographic and environmental space. We then use the models to estimate past [Last Glacial Maximum (LGM) and mid‐Holocene] habitat suitability to compare with previously hypothesized glacial refugia for this species. Environmental niches are statistically significantly different from each other for all pairs of lineages in geographic and environmental space, and these differences cannot be explained by habitat availability within their respective ranges. Together with the inability of most of the lineages to correctly predict the distributions of other lineages, these results support intraspecific ecological differentiation in the bank vole. Model projections of habitat suitability during the LGM support glacial survival of the bank vole in the Mediterranean region and in central and western Europe. Niche differences between lineages and the resulting spatial segregation of habitat suitability suggest ecological differentiation has played a role in determining the present phylogeographic patterns in the bank vole. Our study illustrates that models pooling lineages within a species may obscure the potential for different responses to climate change among populations.

## Graphical abstract

We explore whether phylogeographic lineages of the bank vole originating from different glacial refugia show environmental niche differentiation. We found that niches were significantly different between all pairs of lineages in geographic and environmental space and these differences cannot be explained by habitat availability within their respective ranges, suggesting that ecological differentiation has played a role in determining the present phylogeographic patterns in the bank vole.

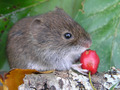

## 1 INTRODUCTION

Environmental niche models correlating species locality records with environmental data to identify conditions suitable for the persistence of the species have been crucial in efforts to understand how past environmental change has impacted species, and how future climate change may affect them as well (Wiens et al., 2009). The default approach of correlative environmental niche modeling (ENM) has long been to use the occurrence data for the whole species within a single model (Smith, Godsoe, et al., 2019). Such studies have provided crucial information about the environmental determinants of species distributions in space and across time, but offer little insight into the role of intraspecific ecological differentiation in the response of species to environmental variation (Chardon et al., 2020). The rate of niche evolution is generally slow, resulting in the tendency for more closely related evolutionary lineages to occupy more similar environments (i.e., niche conservatism; Pearman et al., 2008; Peterson, 1999; Wiens & Graham, 2005). However, it is now well recognized that reduced gene flow, coupled with spatial environmental heterogeneity, can promote the development of local adaptation, which can cause shifts in environmental tolerances between populations of the same species (Sánchez‐García et al., 2015; Serra‐Varela et al., 2015; Shinneman et al., 2016; Smith, Beever, et al., 2019). Consequently, recent ENM studies have begun to explore intraspecific niche variation by separately modeling smaller units within a species range. Such studies indicated that niche differentiation between subspecies and intraspecific phylogenetic lineages may not be uncommon (D’Amen et al., 2013; Gutiérrez‐Rodríguez et al., 2017; Hällfors et al., 2016; Homburg et al., 2014; Ikeda et al., 2017; Jaime et al., 2014; Martínez‐Gordillo et al., 2010; Razgour et al., 2019; Schwalm et al., 2016; Serra‐Varela et al., 2017; Theodoridis et al., 2018). Nevertheless, as intraspecific‐level ENM still represents a small fraction of all ENM studies hitherto conducted (Chardon et al., 2020), the evolutionary and biogeographical significance of ecological niche differences within species remains poorly understood (Pahad et al., 2020).

While undeniably useful for examining patterns of niche evolution that would otherwise be intractable through direct observation or experimentation (Warren, 2012), the correlative ENM comes with several well‐known methodological and conceptual challenges. For example, since the information comes from realized distributions (i.e., constrained by available environmental conditions, dispersal limitations, and biotic interactions) correlative ENM may be more limited in its ability to accurately quantify the niche (Anderson, 2013). Given these inherent limitations, the habitat suitability inferred from correlative ENM results should not be viewed as an absolute prediction of the true fundamental or realized niche, but rather as a proxy for testing hypotheses with respect to niche preferences with regard to the major abiotic conditions experienced by the species (Kearney & Porter, 2009; Kozak et al., 2008; Warren, 2012). Fortunately, as noted by Warren (2012), it is not necessary that the models are perfect or complete estimates of the niche, as even approximate estimates of the niche can be informative for comparative studies.

**FIGURE 1:**
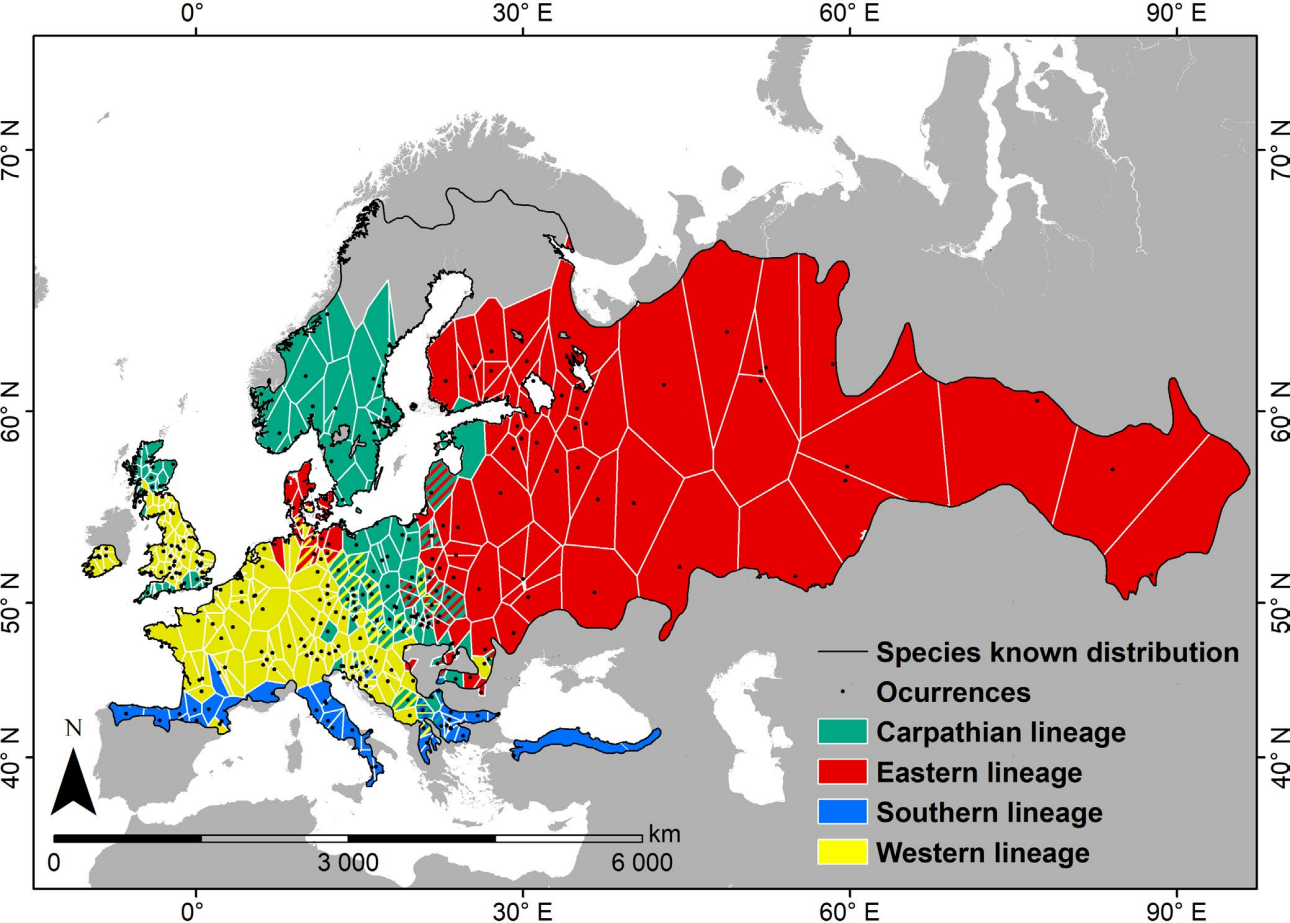
Present distribution of the bank vole phylogeographic lineages estimated by Thiessen polygons for the occurrence data (black dots) and clipped to the bank vole distribution range. The present distribution of the bank vole was derived from the IUCN Red List (Hutterer et al., 2016). Geographic overlap between different lineages is shown with hatched areas

Here, we use ENM to test for intraspecific niche differentiation in the bank vole *Clethrionomys glareolus* (aka *Myodes glareolus*; Kryštufek et al., 2020). As one of the key mammals used in studies of the response of European fauna to climate change following the Last Glacial Maximum (LGM; 22–17 kyr ago; Svendsen et al., 2004), understanding drivers of climate responses in the bank vole provides an important model for other European species (Marková et al., 2020). Many temperate species in Europe were restricted to multiple allopatric refugia during the LGM, which promoted intraspecific divergence and facilitated local adaptation (de Lafontaine et al., 2018). The bank vole has one of the broadest ranges of any European mammal, occurring from the British Isles and northern Spain to western Siberia (Figure 1). It inhabits a diverse range of woodland habitats that include Mediterranean mountain and temperate floodplain forests, boreal and subarctic forests, and a variety of scrub habitats. Despite the general view that much of mid‐ and high‐latitude Europe was largely treeless during the LGM, with woodland refugia restricted to the Mediterranean, increasing fossil evidence shows that bank vole populations survived also in central and western Europe. This includes LGM and late glacial assemblages from the Western Carpathians in Slovakia (e.g., Dzerava skala Cave; Horáček, 2000, 2006) and Poland (Deszczowa and Oblazowa Cave; Nadachowski & Valde‐Nowak, 2015; Sommer & Nadachowski, 2006), from Belgium (Walou Cave; Cordy, 1991), and also from the vicinity of the Ural Mountains and/or western Siberia in Russia (the Pechora basin; Markova et al., 1995; Melnikova et al., 2012).

Previous phylogeographic studies revealed that the bank vole comprises four major evolutionary lineages (Figure 1) inferred to have diverged in separate glacial refugia during the LGM based on their divergence, spatial distribution, and demographic history (Deffontaine et al., 2005; Filipi et al., 2015; Kotlík et al., 2006; Marková et al., 2020). The traditionally recognized south European refugial areas of Iberia, Italy, and the Balkans (Hewitt, 1996) are occupied by endemic populations of a Southern lineage, which shows only limited expansion further north (Bilton et al., 1998; Deffontaine et al., 2005). Instead, the majority of the current bank vole range has been colonized by populations from three widespread lineages: (i) the Carpathian lineage that has been traced to the cryptic refugia in the vicinity of the Carpathians (Kotlík et al., 2006) and nowadays occupying large areas in central and northern Europe (Marková et al., 2020); (ii) a Western lineage that originated from refugia in the vicinity of the Alps and/or western Balkans (Kotlík et al., 2006); and (iii) Eastern lineage that originated in eastern Europe and/or near the Ural Mountains (Deffontaine et al., 2005). It has been suggested that bank vole lineages originating from the different refugia have been exposed to different climate pressures, resulting in intraspecific ecological differentiation, which could explain why the lineages occupy different geographic areas within the present species distribution range (Kotlík et al., 2014). In support of this hypothesis, bank vole populations from the different lineages show divergent tolerances to physiological stress (Kotlík et al., 2014), which may reflect adaptations to environmental conditions (Strážnická et al., 2018).

We use the four bank vole lineages as a first‐order approximation of genetically related populations that are evolutionarily and geographically disjunct and are thus likely to represent adaptive units with specialized environmental tolerances (Chardon et al., 2020; Hällfors et al., 2016). Our primary objective was to assess whether lineages differ in the environmental space they occupy, which would suggest intraspecific ecological differentiation and provide support for a role of local adaptation in the present biogeographical patterns in this species. We also estimate the past (LGM and mid‐Holocene) habitat suitability for the bank vole and for the individual lineages, which we discuss in light of the previously inferred locations of glacial refugia for this and other species.

## 2 MATERIALS AND METHODS

### 2.1 Species occurrences

We compiled a total of 437 occurrence localities for bank vole lineages (Table S1.1). We adopted a different filtering scheme for the species‐level dataset, then for the data split into the different lineages (Anderson et al., 2010; Dungan et al., 2002). For the species‐level dataset, we used a 2‐step filtering scheme as there was a need to distribute the records as evenly as possible due to the comparably lower sampling effort for the Siberian portion of the bank vole distribution range occupied by Eastern lineage. We first discarded the records matching the same pixel on the 30‐arc‐second resolution bioclimatic layer (see below), retaining only one record per pixel. We then generated a 5° × 5° grid in ArcMap, v.10.6 (Esri, 2018), and retained a maximum of 5 records per cell in order to adjust for geographic biases due to uneven sampling effort across the bank vole range (Vega et al., 2010; Wisz et al., 2008). When modeling individual lineages, it was no more necessary to distribute the records evenly across the entire bank vole range. Instead, it was desirable to retain a maximum number of occurrences for each lineage. Therefore, within each of the four lineage datasets we only filtered out records that were within 10 km (0.09°) from one another to minimize spatial autocorrelation and avoid the introduction of biases due to sampling at multiple adjacent localities (Ruiz‐Luna et al., 2017). The bank vole distribution polygon was obtained from the IUCN Red List (Hutterer et al., 2016) and is useful for an approximate delineation of the bank vole range for the purpose of our study (Figure 1). For visualization purposes and for further analyses (Section 2.4), the distribution range of each lineage was estimated from the occurrence data available for that lineage in ArcMap by creating Thiessen polygons, that is, polygons whose boundaries define the area that is closest to each point relative to all other points (also known as Voronoi tessellation cells) (Horton, 1917), clipped to the extent of the bank vole distribution range (Figure 1).

### 2.2 Environmental data

Raster layers representing 19 bioclimatic variables were downloaded from the WorldClim (www.worldclim.com) v.1.4 dataset (Hijmans et al., 2005), at 30‐arc‐second resolution, and were clipped to the boundaries of the study area (see below). The bioclimatic variables from WorldClim are a widely employed set of environmental data layers for ENM because of their high resolution, global coverage, and availability for both present and past climate scenarios. To account for climate modeling uncertainties (Schorr et al., 2012), two general circulation models (GCMs) of past climate, CCSM4 (Gent et al., 2011) and MIROC‐ESM (Watanabe et al., 2011), were used for the LGM (about 22 kyr ago) and mid‐Holocene (about 6 kyr ago), which predict a relatively colder and dryer (CCSM4) versus warmer and wetter (MIROC‐ESM) LGM environments over the Northern Hemisphere (Nicolas et al., 2017).

### 2.3 Environmental Niche modeling

The ENM was performed using the maximum entropy approach implemented in MaxEnt, v.3.4.1 (Phillips et al., 2004), which does not require absence data and is among the least sensitive to sampling biases when studying large geographic areas (Qiao et al., 2015).

Two different sets of predictor variables were used to ascertain the robustness of the results (Araújo et al., 2019). Both sets were selected based on a reiterative jackknife procedure of model construction and stepwise removal of the least contributing variables (Zeng et al., 2016), but each used a different dataset and metric (i.e., training gain or test gain) to measure the contribution of the variables to the model. After removing the uninformative variables by the jackknife procedure, the final set of variables was produced in each case by removing one variable from each pair of correlated variables, based on a correlation matrix of the climate layers (cut‐off of *r* ˂ 0.8; Merow et al., 2013). The first set of predictors (Set 1) was selected based on the training gain using the species‐level occurrences as the training dataset, and it contained Mean Diurnal Range (BIO 2), Temperature Annual Range (BIO 7), Mean Temperature of Wettest Quarter (BIO 8), Mean Temperature of Driest Quarter (BIO 9), Precipitation Seasonality (BIO 15), Precipitation of Wettest Quarter (BIO 16), Precipitation of Driest Quarter (BIO 17), and Precipitation of Warmest Quarter (BIO 18). The second set (Set 2) was selected using Western Siberia as an independent test area where we generated a grid with cell size of 0.5° × 0.5° (*n* = 948 cells) within the outline of the bank vole distribution range and used the cell centroids as the test data. Western Siberia is considered as an area with the current climatic conditions resembling those in Europe during the LGM, and its use as the test area helped ensure the transferability of the models (Fløjgaard et al., 2009) and reduce any bias from the limited number of bank vole occurrences available for the Siberian part of its distribution range (Vega et al., 2010). The variables included in Set 2 based on the test gain were Isothermality (BIO 3), Mean Temperature of Warmest Quarter (BIO 10), Precipitation of Wettest Quarter (BIO 16), and Precipitation of Warmest Quarter (BIO 18).

Niche models were built independently with Set 1 and Set 2. A total of 50 replicates of each model were generated by the subsampling method in MaxEnt, which randomly selected 25% of the occurrence points reserved as test data (Phillips et al., 2004). Subsequently, the estimates are based on the overall mean of the replicates. To minimize the possible effect of inadequate representation of the environmental background (Guevara et al., 2018), 1,000,000 background points were used. Default values were used for the other parameters, as recommended when comparing models at different evolutionary levels (i.e., species and intraspecific lineages) and with different sampling efforts (Merow et al., 2013; Phillips & Dudík, 2008).

Our study area encompasses the bank vole distribution range and surrounding areas, covering Europe, western Siberia, the Anatolian Peninsula, and the Caucasus. We had no a priori reasons to exclude specific parts of Europe as inaccessible to colonization by the different bank vole lineages. Geographic distributions of the lineages do not align with major geographic barriers, and it is known that European small mammals responded individualistically to the end‐glacial warming in terms of the contribution of the different refugia to colonization of the different parts of Europe (with some species colonizing most of Europe from a single refugium), indicating that the present phylogeographic patterns are, to large extent, the product of species’ adaptive niches and habitat availability (Michaux et al., 2005). Furthermore, the evidence shows that during the end‐glacial colonization bank vole lineages occupied areas where they are no longer present, and which are currently occupied by other lineages (Figure 1; Kotlík et al., 2018; Searle et al., 2009). Thus, the study area encompassing the entire distribution range of the bank vole should reasonably represent the broader background landscape likely to have been “tested” by various lineages for suitability, even though not presently occupied by them (Barve et al., 2011).

To evaluate the performance of the models, we first calculated the average area under the receiver operating characteristic (AUC) curve for the test data (Guisan & Zimmermann, 2000), including 95% confidence intervals. We then calculated the partial AUC (pAUC) ratio using Niche Tool Box (Osorio‐Olvera et al., 2020; Peterson et al., 2008) and the sensitivity index based on the omission rate at the minimum training presence (the lowest value among all training records; MTP) and 10% training presence (the value below which 10% of all training records fall) thresholds (Kumar et al., 2015).

The models were projected to the CCSM4 and MIROC‐ESM bioclimatic layers for the LGM and mid‐Holocene. The logistic format and ascii type were used to generate the raster output. Response curves were constructed to illustrate the effect of each variable on the modeled habitat suitability. Three different thresholds were applied to generate binary maps of suitable habitat: the minimum training presence, the fifth percentile training presence, and the 10th percentile training presence (Liu et al., 2005). To assess the ability of the model constructed for each lineage to predict the known distribution range of the other lineages, we calculated how many occurrences available for each lineage were successfully predicted by the binary maps (suitable/unsuitable habitats) constructed for each other lineage (Peterson, 1999), applying the three thresholds in order to account for the sensitivity of binary presence–absence predictions to the threshold choice (Li & Guo, 2013). A composite prediction from the models constructed for the individual lineages (Chardon et al., 2020) was generated by merging the binary maps across the lineages for each threshold. As a summary of the results across the four predictor (Set 1 and Set 2) and GCM (CCSM4 and MIROC‐ESM) combinations, frequency ensemble maps were generated showing the number of models predicting the presence of a given lineage in any particular area based on the MTP threshold, separately for the mid‐Holocene and LGM (Araújo & New, 2007).

### 2.4 Niche comparison

Niche breadth was quantified by Levins’ metric (*B*) on a relative scale of 0 to 1, with higher values indicating broader niches (Levins, 1968). Niche overlap was described by Schoener's *D* (Schoener, 1968; Warren et al., 2008) and a modification of Hellinger's *I* distances (Warren et al., 2008) on a scale of 0 (no overlap) to 1 (identical niche models). The breadth metric measures the uniformity of the distribution of suitability scores for a model, while niche overlap metrics measure the similarity of the distribution of suitable environment for a pair of models (Warren et al., 2021). The calculations were performed independently for models built with each predictor set (Set 1 and Set 2).

Niches were compared by using two different approaches. First, we calculated niche breadth and overlap based on habitat suitability predicted by the niche models for the geographic space (*G*‐space), which is represented by all the combinations of values of bioclimatic variables observed across the study area (Warren et al., 2008). Measurements of niche similarity in *G*‐space are suitable to evaluate the potential for the different lineages to occupy the existing habitats on European landscape, but they can be somewhat misleading if the availability of habitat types on the landscape is strongly biased and if the environmental tolerances of the lineages are not equally represented in the geographic space (Broennimann et al., 2012; Brown & Carnaval, 2019; Warren et al., 2019). Therefore, as a second approach, we applied a novel method for niche model comparison in a sample from continuous multidimensional space of environmental variables (*E*‐space) generated by Latin hypercube sampling with a chunk size of 100,000 points and tolerance of 0.00001 (Warren et al., 2019). The calculations in *G*‐space were performed with ENMTools 1.4.4 (Warren et al., 2010) and those in *E*‐space using the env.breadth and env.overlap functions of the ENMTools R package (Warren et al., 2021). To evaluate whether the niche overlap is statistically significant, we applied two randomization tests. The first, niche identity (or equivalency) test evaluated whether the niche models for two lineages can be considered identical based on a null distribution generated by randomizing the occurrences between the lineages (Warren et al., 2008). The hypothesis that the niches are identical is rejected when the empirical *D* or *I* value is significantly (at the 0.05 level) lower than expected from the null distribution (Warren et al., 2008, 2010). The identity tests were performed separately for niche comparison in *G*‐space and *E*‐space, by using ENMTools 1.4.1 and the ENMTools R function identity.test, respectively. The second, background similarity test evaluated whether the niches for two lineages are more similar, or different, than expected by chance, given the environmental differences between the regions in which the lineages occur (Warren et al., 2008, 2019). The test compared the overlap between the observed niches of two lineages with a null distribution expected between one lineage and a sample of random occurrences for another lineage (Warren et al., 2008). In case the observed value of niche overlap is significantly lower (indicating more different niches than expected by chance) or higher (indicating more similar niches than expected by chance) than expected from 100 pseudoreplicates, the null hypothesis of the difference between the lineages being no different than expected based on environmental differences between their distribution ranges is rejected. The random occurrences for each lineage were generated within the Thiessen polygons of its distribution range (Figure 1). The similarity test of niche overlap in *G‐*space was calculated with ENMTools 1.4.1 and that in *E*‐space with the ENMTools R function background.test.

Finally, to visualize the differences between niches occupied by the bank vole and each of its lineages in *G*‐space and *E*‐space, we performed a principal component analysis (PCA) on the set of climate rasters used to build the models, separately for Set 1 and Set 2, and plotted the occurrence data together with the background points and a 100,000‐point sample of the multidimensional *E*‐space (Warren et al., 2019).

## 3 RESULTS

### 3.1 Model performance

The filtered occurrence dataset for the species‐level ENM consisted of 210 records. Those for the individual lineages included 107 records for the Carpathian lineage, 85 records for the Eastern lineage, 36 records for the Southern lineage, and 138 occurrences for the Western lineage (Table 1 and Figure 1).

**TABLE 1:** Performance of ecological niche models build using two different sets of climatic variables (Set 1 and Set 2), evaluated by the average test area under the curve (AUC), test AUC confidence intervals, partial AUC ratio calculated at 0% omission rate (pAUC), and sensitivity index based on the omission rate at the minimum training presence (OR 0%) and 10 percentile training presence (OR 10%) thresholds for 50 replicates; and niche breadth quantified by Levins’ inverse concentration metric in geographic (*G*) and environmental (*E*) space

Predictors	Model	All occurrences	Filtered occurrences	Test AUC	Confidence interval	pAUC	OR 0%	OR 10%	Niche breadth *G*‐space	Niche breadth *E*‐space
Set 1	*C. glareolus*	437	210	0.82	±0.007	1.66	0	0.097	0.55	0.42
	Carpathian	145	107	0.92	±0.003	1.88	0	0.100	0.24	0.12
	Eastern	113	85	0.85	±0.008	1.73	0	0.094	0.46	0.10
	Southern	37	36	0.96	±0.005	1.94	0	0.074	0.14	0.17
	Western	194	138	0.94	±0.002	1.89	0	0.096	0.18	0.14
Set 2	*C. glareolus*	437	210	0.80	±0.007	1.61	0	0.097	0.76	0.45
	Carpathian	145	107	0.90	±0.004	1.82	0	0.099	0.31	0.29
	Eastern	113	85	0.83	±0.009	1.68	0	0.094	0.59	0.17
	Southern	37	36	0.95	±0.006	1.92	0	0.074	0.23	0.27
	Western	194	138	0.94	±0.002	1.60	0	0.096	0.18	0.43

The effect of each variable included in Set 1 on the regularized training gain indicated that Precipitation of Driest Quarter showed the highest percent contribution to the species‐level models, Temperature Annual Range to models built for the Carpathian and Western lineages, Mean Temperature of Driest Quarter for the Eastern lineage, and Mean Diurnal Range for the Southern lineage. In Set 2, Isothermality had the highest percent contribution to all models. The response curves of the variables for the bank vole and the individual lineages are shown in Figure S2.1.

All models performed significantly better than expected by chance. Model AUC values ranged from 0.80 to 0.96 with the 95% confidence intervals of ±0.002 to ±0.009 (Table 1), reflecting excellent (species‐level and Eastern lineage) and outstanding (Carpathian, Western, and Southern lineage) model performance, respectively (Araújo et al., 2005). All models also performed better than random according to pAUC values >1 (Table 1; Kumar et al., 2015; Peterson et al., 2008). Finally, the model omission rates were equal to or lower than expected (Radosavljevic & Anderson, 2014), such that the test sensitivity was 0 at the 0% training omission and ranged from 0.07 to 0.10 at the 10% training omission (Table 1).

### 3.2 Present habitat partitioning

**FIGURE 2:**
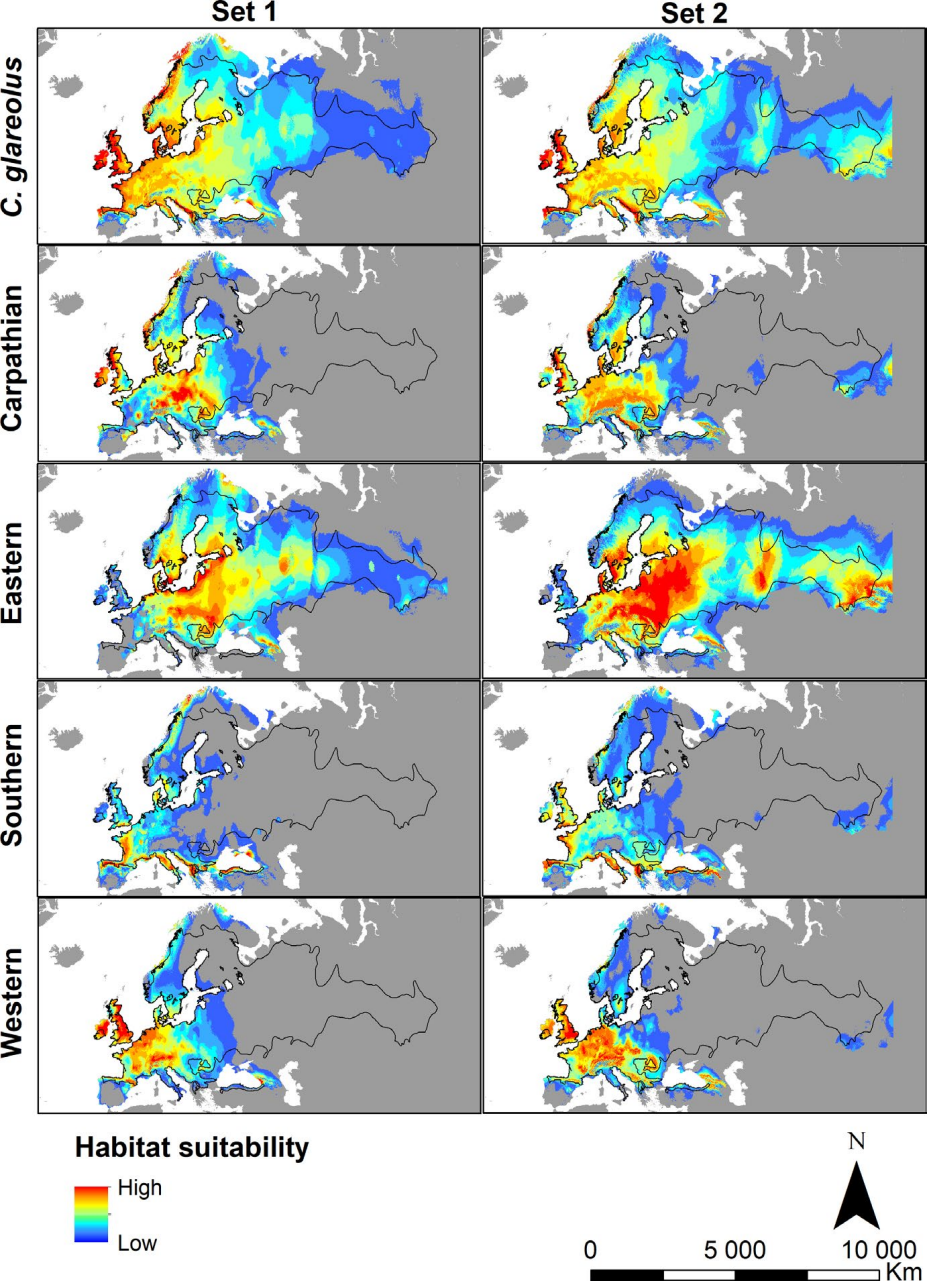
Current habitat suitability for the bank vole and each of the four phylogeographic lineages predicted using two different sets of climatic variables (Set 1 and Set 2). The boundary of the bank vole distribution range (Hutterer et al., 2016) is represented by the black polygons

For both sets of variables (Set 1 and Set 2), the geographic distribution of habitat suitability for the bank vole under the current climate closely approximates the present distribution range of the species (Figure 2). Applying different thresholds had mainly an effect in West Siberia, where the more stringent thresholds (5% and 10%) resulted in a more restricted habitat suitability than expected from the current distribution range of the species (Figure S2.2). Applying the least stringent threshold (MTP), on the other hand, predicted habitat suitability above the northern edge of the bank vole range, especially with Set 1 (Figure S2.2). In Asia, all thresholds and both sets of variables predicted an area of high habitat suitability in the vicinity of the Caucasus, which is outside the known bank vole distribution range (Figure S2.2).

Although the composite prediction from the models constructed for the individual lineages (Figure S2.3–S2.4) closely matches that from the species‐level models (Figure 2 and Figure S2.2), there are large differences in the habitat suitability for the different lineages. In general, areas of habitat suitability for each lineage encompass its present distribution range (Figure 1), albeit with some overprediction (Figure 2), especially when the least stringent threshold is applied (Figure S2.2). Habitat suitability for the Carpathian lineage is predicted over a large part of central Europe to the north and west of the Carpathian Basin, in southern and eastern Scandinavia and in Great Britain (Figure 2, Figure S2.2). The habitat suitability for this lineage is predicted to extend westward up to the Atlantic Coast and English Channel, although populations from the Carpathian lineage are presently absent from much of western continental Europe (Figure 1). For the Eastern lineage, habitat suitability is predicted in central and eastern Europe as far as the Ural Mountains and beyond into West Siberia. Large areas of habitat suitability are also identified in southern Fennoscandia (Sweden and Norway), where the Eastern lineage presently does not occur (Figure 2). Similar to the species‐level model, the ENM for the Eastern lineage resulted in an underprediction of habitat suitability in West Siberia when the stringent thresholds were applied (Figure S2.2). As expected, the highest habitat suitability for the Southern lineage was predicted along the three Mediterranean Peninsulas and in southern and western France. Areas of high habitat suitability for this lineage are also predicted further north, along the west coast of France and Great Britain and even Scandinavia. For the Western lineage, habitat suitability is mostly restricted to western Europe, largely matching the present distribution of the lineage (Figure 1). As for the species models, an area of habitat suitability for all lineages is identified in the Caucasus (Figure 2).

In general, the habitat suitability for Carpathian, Southern, and Western lineages responded positively to high precipitation levels. In contrast, for Eastern lineage, high habitat suitability was predicted mostly in drier climates, with a strong negative influence of precipitation of the wettest (BIO 16) and driest (BIO 17) quarters (Figure S2.1).

The models for the individual lineages showed varying abilities to predict the occurrences of the other lineages (Table S2.1). For example, with Set 1, the model for the Southern lineage predicted only 5 (5.9%), 11 (12.9%), and 16 (18.8%) of 85 occurrences of the Eastern lineage when considering the 10%, 5%, and MTP threshold, respectively, and 18 (16.8%), 20 (18.7%), and 35 (32.7%) of 107 occurrences of the Carpathian lineage when applying the same thresholds. On the other hand, the models for the Carpathian and Western lineages were more successful in predicting each other's distributions, with up to 100% of Western lineage occurrences (138) predicted by the model for the Carpathian lineage with Set 2, when considering the least stringent (MTP) threshold (Table S2.1).

### 3.3 Past habitat suitability

**FIGURE 3:**
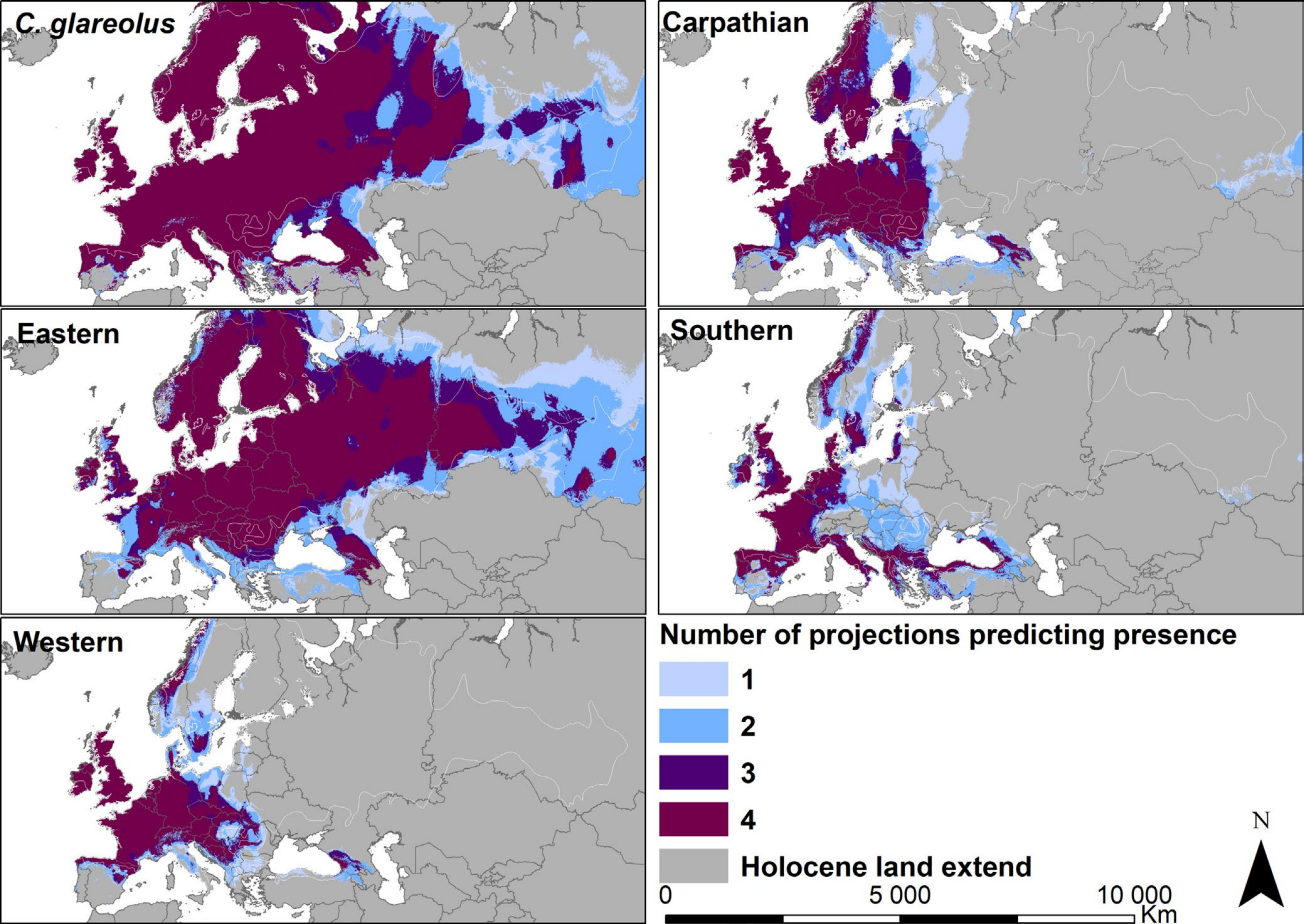
Mid‐Holocene habitat suitability ensemble maps for the bank vole and each of the four phylogeographic lineages, with increasing numbers indicating areas where multiple projections predict the presence. The boundary of the current bank vole distribution range (Hutterer et al., 2016) is represented by the white polygons

**FIGURE 4:**
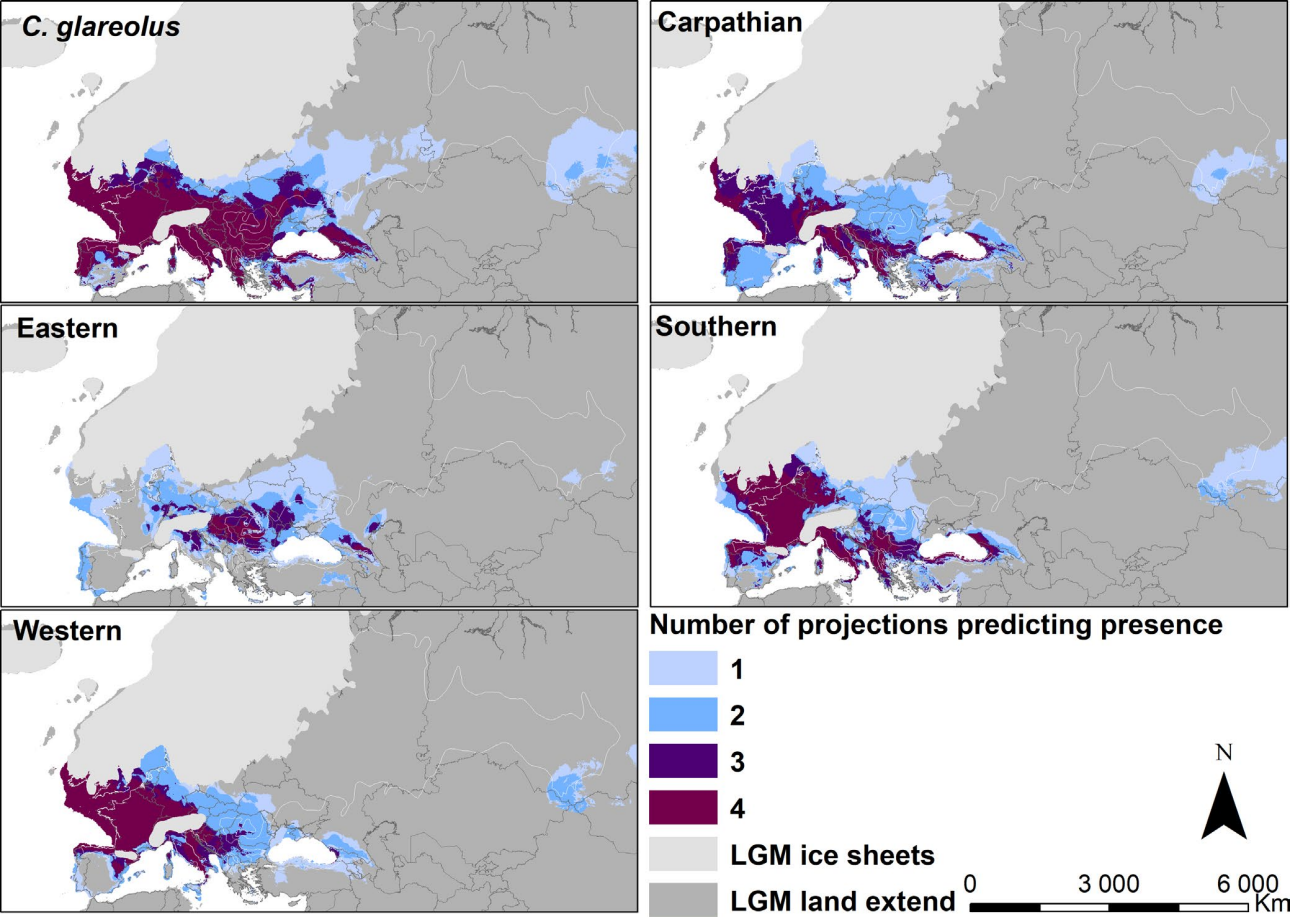
Last Glacial Maximum (LGM) habitat suitability ensemble maps for the bank vole and each of the four phylogeographic lineages, with increasing numbers indicating areas where multiple projections predict the presence. The boundary of the current bank vole distribution range (Hutterer et al., 2016) is represented by the white polygons

In the mid‐Holocene, the geographic distribution of each lineage's habitat suitability was similar to its present distribution (Figure 3 and Figure S2.5). However, habitat suitability was likely more restricted during the LGM, primarily in eastern Europe and western Siberia (Figure 4). According to all four combinations of the past climate models (CCSM4 and MIROC‐ESM) and sets of variables (Set 1 and Set 2), large areas of high habitat suitability for the bank vole existed on the three Mediterranean Peninsulas of Iberia, Italy, and the Balkans, in the west of Europe (present France), in the Carpathian Basin and around, and north of the Black Sea, including the Caucasus (Figure 4). Habitat suitability is predicted to have extended onto the exposed shelf along the Atlantic coast, northward up to the Doggerland landmass in the present North Sea (Figure 4). Models using Set 2 further predict isolated areas of high habitat suitability in western Siberia in the vicinity of the Ural and Altai mountains (Figure S2.6).

Models for the individual lineages produced considerably overlapping predictions of habitat suitability during the LGM (Figure 4), with Set 2 generally yielding larger areas than Set 1, especially with the CCSM4 layers (Figure S2.6). For the Western, Carpathian, and Southern lineages, models suggest high habitat suitability in the Mediterranean region, the Carpathian Basin, and the west of continental Europe, including the exposed Atlantic shelf and Doggerland (Figure 4). High habitat suitability for these three lineages is also consistently predicted near the Caucasus and along the Black Sea shelf (Figure 4). Predictions for the Eastern lineage identify much more restricted habitat suitability (Figure 4), especially with Set 1 and when more stringent thresholds are applied (Figure S2.6), with high probability conditions largely restricted to the Carpathian Basin, vicinity of the Alps and north of the Black Sea (Figure 4). Highly supported areas of habitat suitability for all four lineages are predicted to have existed in the vicinity of the Alps, southern Carpathian Basin, and Caucasus (Figure 4).

### 3.4 Niche comparison

Broader niche estimates were obtained for the entire species than for any of the four bank vole lineages, in both *G*‐space and *E*‐space (Table 1). Of the four lineages, the Eastern lineage has the broadest niche in *G*‐space, but the narrowest in *E*‐space. In contrast, the Southern and Western lineages have the narrowest niches in *G*‐space, but are broadest in *E*‐space (Table 2).

**TABLE 2:** Tests of niche identity between bank vole lineages calculated based on niche models built with two different sets of climatic variables (Set 1 and Set 2)

Predictors	Lineage A	Lineage B	Lineage A versus lineage B			
			*G*‐space		*E*‐space	
			*D*	*I*	*D*	*I*
Set 1	Carpathian	Eastern	0.53**	0.78**	0.34*	0.54**
		Southern	0.42**	0.71**	0.10**	0.22**
		Western	0.68**	0.92**	0.30**	0.49**
	Eastern	Southern	0.25**	0.49**	0.08**	0.19**
		Western	0.39**	0.66**	0.15**	0.30**
	Southern	Western	0.48**	0.76**	0.10**	0.25**
Set 2	Carpathian	Eastern	0.52**	0.81**	0.39**	0.65**
		Southern	0.62**	0.87**	0.30**	0.53**
		Western	0.66**	0.91**	0.48*	0.77*
	Eastern	Southern	0.37**	0.68**	0.10**	0.23**
		Western	0.30**	0.61**	0.21**	0.43**
	Southern	Western	0.63**	0.86**	0.29**	0.53**

Note

Niche overlap, quantified by Schoener's *D* and Hellinger's *I*, is evaluated in geographic (*G*) and environmental (*E*) space. A significant identity test indicates that lineages have developed measurable differences in niche occupancy.

Niches significantly different at *p ≤ .05 and **p ≤ .01.

Niche overlap between the lineages tends to be higher in *G*‐space than in *E*‐space for both measures (Table 2, 3). In *G*‐space, the lowest *D* values were obtained between niche models of the Eastern and Southern lineages when using Set 1 and between the Eastern and Western lineages with Set 2 (Table 2). The lowest overlap in *E*‐space is observed between the Eastern and Southern lineages for both Set 1 and Set 2 models. The highest niche overlap in *G*‐space is observed between the Carpathian and Western lineages. In *E*‐space, the Carpathian lineage showed the highest niche overlap with Eastern lineage when modeled with Set 1 and with Western lineage with Set 2 (Table 2). The individual lineages showed only partial niche overlap with the species‐level model in both *G*‐space (*D* of 0.42–0.73) and *E*‐space (*D* of 0.25–0.55), with the Eastern lineage showing substantially higher overlap than the other lineages in *G*‐space, but the lowest in *E*‐space (Table S2.2).

**TABLE 3:** Background similarity tests calculated based on niche models built using two different sets of climatic variables (Set 1 and Set 2). Niche overlap is measured by Schoener's *D* and Hellinger's *I* in geographic (*G*) and environmental (*E*) space (see Table 2)

Predictors	Lineage A	Lineage B	Lineage A versus lineage B				Lineage B versus lineage A			
			*G*‐space		*E*‐space		*G*‐space		*E*‐space	
			*D*	*I*	*D*	*I*	*D*	*I*	*D*	*I*
Set 1	Carpathian	Eastern	Similar**	Similar**	NS	NS	NS	NS	NS	NS
		Southern	Similar*	Similar*	Different**	Different**	Similar*	NS	Different**	Different**
		Western	Similar**	Similar**	NS	NS	Similar**	Similar**	NS	NS
	Eastern	Southern	NS	NS	Different**	Different**	Similar**	Similar**	Different**	Different*
		Western	Similar**	Similar**	NS	Different*	Similar**	Similar**	Different**	Different*
	Southern	Western	NS	NS	Different**	Different**	NS	NS	Different**	Different**
Set 2	Carpathian	Eastern	Similar**	Similar**	NS	NS	Different*	NS	NS	NS
		Southern	Similar**	Similar**	NS	Different*	Similar**	Similar**	Different**	Different*
		Western	Similar**	Similar**	NS	NS	Similar**	Similar**	NS	NS
	Eastern	Southern	Similar**	NS	Different**	Different**	Similar**	Similar**	Different**	Different**
		Western	Similar**	Similar**	Different**	Different**	Similar**	Similar**	Different**	Different**
	Southern	Western	Similar**	Similar**	Different*	Different**	NS	NS	Different**	Different**

*p ≤ .05; **p ≤ .01; NS, not significant; Different, niches significantly more different than expected based on available habitat differences; Similar, niches significantly more similar than expected based on available habitat differences.

A significant background test indicates that the observed niche differences between lineages are a function of habitat selection and/or suitability rather than an artifact of the underlying environmental differences between the suite of habitats experienced by the two lineages (Warren et al., 2008).

The identity tests rejected the niche equivalency hypothesis for all pairs of lineages, using either Set 1 or Set 2, or *D* or *I* metrics (Table 2), showing that the lineages occupy different portions of *G*‐space and *E*‐space (Figure S2.7). For the majority of the comparisons, the background similarity test rejected the hypothesis that the niche differentiation is explained by environmental differences between the habitats available within the present ranges of the lineages (Table 3). For most of the significant comparisons in *G*‐space (34 out of 35), the background similarity test indicated that niches of the lineages are more similar than expected based on the available environmental conditions (Table 3). A contrasting pattern is observed when niches are compared in *E*‐space, with niches more different than expected in all 30 significant comparisons (Table 3).

## 4 DISCUSSION

The bank vole has one of the most complex phylogeographical patterns of all European mammals studied to date (Marková et al., 2020), and here, we use ENM to elucidate environmental variation among the bank vole phylogeographic lineages. We demonstrate significant niche differences between the four lineages, which suggests habitat suitability as the mechanism contributing to the spatial segregation of these lineages and thus ultimately to the present biogeographical (phylogeographical) patterns in the bank vole. Model projections further support in situ LGM survival of the bank vole in central and western Europe (Kotlík et al., 2006). The bank vole is one of the key mammals used in studies of the response of European fauna to climate change following the LGM (Deffontaine et al., 2005; Filipi et al., 2015; Marková et al., 2020). Therefore, understanding the role of ecological niche variation in relation to phylogeographic differentiation in the bank vole provides an important model for understanding and predicting these patterns and processes in other species.

### 4.1 Niche differentiation in the bank vole

The results show that the four bank vole lineages have developed measurable differences in niche occupancy and any attempt to predict the niche characteristics of one lineage from that of another lineage would thus be inadequate for this species. The background similarity tests indicated that the observed differences are likely a function of habitat selection and/or suitability rather than simply an artifact of differences in habitats available to the lineages within their respective geographic ranges (Warren et al., 2008). Niche divergence between the bank vole lineages is indicated by the fact that niches are more different across a multidimensional *E*‐space than expected. Although still significantly different from each other, the lineages are more similar when compared across *G*‐space than would be expected based on available habitat differences, likely due to the fact that the environmental combinations available across Europe represent a nonrandom subset of the potential environments (Warren et al., 2019). This result is not unexpected as a high degree of niche similarity (conservatism) is predicted between intraspecific lineages or closely related species, as the consequence of recent common descent and slow rate of niche evolution (Peterson, 1999; Wiens & Graham, 2005). Our results are congruent with other studies suggesting that closely related lineages could be more similar than expected given available habitats and yet still be sufficiently differentiated in their environmental tolerances that their niches are best modeled by splitting (Hu et al., 2016; Smith, Godsoe, et al., 2019). For example, a single species model might not describe well the niche of a particular intraspecific lineage that is adapted to a specific set of climatic conditions (Pearman et al., 2010). Alternatively, a species model could overestimate the species‐level response to climate change when, in fact, only a few populations within the species could exhibit such a response. Finally, a lineage occupying a small fraction of a species distribution range might make little contribution to a species‐level model, but may be critically important for the response of species to changing climate, potentially representing the surviving part of the species (Pearman et al., 2010).

### 4.2 Geographic and ecological partitioning

The spatial segregation of habitat suitability between lineages, together with the inability of most of the lineages to correctly predict the occurrences of other lineages, supports the niche differentiation indicated by the identity and similarity tests. The geographic overlap between the lineages in certain areas (Figure 1) is consistent with some overlap in their niches (Table 2). Although our results support the common expectation that genetic lineages best explain variation in climate relationships across the species’ distribution range (Chardon et al., 2020), such relationships need to be further confirmed with direct experimental work. For example, nuclear introgression among the lineages may be blurring lines of local adaptation (Horníková et al., 2021), or the genetic subdivisions used here may be too coarse to fully capture heterogeneity in bank vole's responses to climate (Smith, Beever, et al., 2019). The fact that the models tend to predict somewhat broader geographic distribution of habitat suitability for each lineage, compared with its present distribution range, suggests that lineages may not be occupying all areas with suitable conditions that meet their niche requirements (Yackulic, 2017). It is unlikely that biotic factors in an area (e.g., competition with other species) would affect niches of only some bank voles lineages but not others (Brown & Carnaval, 2019). Therefore, we propose that the niches of any given lineage are likely constrained because the niches of the other lineages predict higher habitat suitability values within the range of the given lineage.

The similarity of spatial predictions obtained with the different sets of variables gives added robustness to these results (Figure 2). Using variables selected with Siberia as an independent test zone (Set 2) improved the model projections in the eastern part of the bank vole distribution range, although some underprediction is likely attributable to the low number of records from that region (Figure 2). Interestingly, the models with both sets of variables for all lineages predicted areas of high habitat suitability in the Caucasus, where bank vole presence has not been recorded. It is notable that previous ENM studies suggested habitat suitability in the Caucasus for various European small mammals presently absent from that area. These findings suggest that other mechanisms (e.g., competition with other species or geographic barriers to dispersal; Fløjgaard et al., 2009; Vega et al., 2010) may be in operation in this region. For example, it is possible that the open habitat of the Pontic steppe on the North Caucasus restricts the southward dispersal of woodland species such as the bank vole. Similarly, the habitat suitability overprediction for different bank vole lineages in northern Scandinavia may reflect competition with the northern red‐backed vole (*Clethrionomys rutilus*), which replaces the bank vole throughout the northern taiga and forest‐tundra zones of Eurasia (Marková et al., 2020).

Of the four lineages, the Eastern lineage has the largest distribution and niche breadth in *G*‐space, but the narrowest niche in *E*‐space. Populations with narrow fundamental niches that occupy conditions common in a particular region may have low niche breadth in *E*‐space but high niche breadth when models are projected to *G*‐space (Warren et al., 2019). The habitat suitability for the Eastern lineage was related to drier conditions, and therefore, the contrasting estimates for the Eastern lineage might reflect its broad distribution across the homogeneous landscape of eastern Europe and western Siberia, which is characterized by a continental climate (Kottek et al., 2006). In contrast, the Southern and Western lineages, which occupy comparably smaller areas in southern and western Europe, appear to tolerate broad ranges of environmental conditions across more heterogenous landscapes that encompass a variety of temperature and precipitation regimes (Kottek et al., 2006).

### 4.3 LGM survival

The broad distribution of LGM habitat suitability across unglaciated Europe (Figure 4) reinforces fossil and molecular evidence that the species was not constrained to the Mediterranean region during the LGM (Deffontaine et al., 2005; Horáček, 2000; Kotlík et al., 2006; Nadachowski & Valde‐Nowak, 2015). The models indicate that the southern refugial areas in the Mediterranean peninsulas of Iberia, Italy, and the Balkans had climates within the niche requirements of the Southern, Carpathian, and Western lineages (Figure 4). However, habitat suitability for all four lineages is also predicted for large areas in the Carpathian Basin, in the vicinity of the Alps and along the western coast of Europe (Figure 4). These findings are consistent with the survival of bank vole populations in cryptic refugia in central and western Europe, such as the Carpathians in Slovakia and Poland (Horáček, 2000; Sommer & Nadachowski, 2006) and in Belgium (Cordy, 1991). Our results are thus congruent with earlier ENM studies that predicted broad LGM habitat suitability from species‐level models for the bank vole and some other small mammals such as the field vole (*Microtus agrestis*) (Fløjgaard et al., 2009) and pygmy shrew (Vega et al., 2010). The existence of wooded extra‐Mediterranean refugia was further supported by ENM for European trees, which indicated suitable LGM climate for various boreal and temperate species in central and eastern Europe (Svenning et al., 2008).

While the evidence for LGM survival of the bank vole and other species in non‐Mediterranean Europe is now compelling, care must be taken when interpreting the past habitat suitability estimated with ENM. Low atmospheric CO_2_ concentrations during the LGM probably resulted in patchy forest occurrence, compared with climate alone, and forests would be restricted to more moist habitats in the landscape (Ramstein et al., 2007). Therefore, woodland‐associated species such as the bank vole probably had more restricted and patchy distribution ranges within the areas where the LGM climate was suitable for them than predicted by ENM (Fløjgaard et al., 2009). For example, a pollen analysis of the LGM landscape in the Carpathian Basin showed that lowlands were dominated by dry steppe with wet and mesic grasslands occurring in the river floodplains, with forest patches or scattered trees restricted to river valleys, on north‐facing hillslopes, and at moister sites of the loess plateaus (Magyari et al., 2013). Therefore, although the ENM evidence indicates that climates conducive to survival of the bank vole existed across broad areas in the Mediterranean and more northerly Europe, the species likely survived in more restricted refugia, in a form of geographically isolated populations (Stewart et al., 2010), which contributed to the intraspecific diversification inferred by phylogeographic studies (Deffontaine et al., 2005; Marková et al., 2020).

The LGM models for each lineage show strong prediction of the previously suggested refugial area for that lineage (Deffontaine et al., 2005; Kotlík et al., 2006), for example, along the Mediterranean coast for the Southern lineage and in the Carpathian Basin for the Carpathian lineage (Figure 4 and Figure S2.6). The overprediction and overlap between LGM habitat suitability for the different lineages (Figure 4) are consistent with the observed niche overlap between lineages and their origin from a common pre‐LGM ancestor (Deffontaine et al., 2005). The Eastern lineage is the only lineage, where our models predict much restricted LGM habitat suitability, with high probability conditions centered on the Carpathian Basin and the northern Black Sea region (Figure 4). These results for the Eastern lineage may reflect adaptation to different climates than in the other lineages. However, we cannot exclude some biases in the representation of the climatic niche for this lineage due to the smaller record set from a portion of its distribution range (Mcguire & Davis, 2014), despite using western Siberia as a test area (Set 2 of variables). This could also explain why glacial refugia in the vicinity of the Ural Mountains suggested by the fossil record (Markova et al., 1995; Melnikova et al., 2012) were not identified by our models (Figure 4).

### 4.4 Ecological differentiation and bank vole biogeography

Evidence from phylogeographic studies indicated that bank vole lineages originated as a result of divergence in multiple glacial refugia during the LGM (Deffontaine et al., 2005; Kotlík et al., 2006). The differences in ecological niche associated with the different lineages revealed in this study could help explain why the lineages presently occupy different geographic areas within the bank vole distribution range. It has been suggested that the phylogeographic patterns currently observed in many temperate species may represent an intermediate state of a spontaneous diffusion process, after the removal of a geographic barrier imposed by isolation in LGM refugia (Hofreiter et al., 2004). However, the niche differences between bank vole lineages and the resulting spatial difference in habitat suitability provide support for the idea that ecological differentiation may also play a role in generating and maintaining phylogeographic patterns, even in the absence of strong geographic barriers (i.e., “adaptive phylogeography”; Kotlík et al., 2014). Although the habitat suitability inferred from correlative ENM is not an absolute prediction of the true fundamental or realized niche of the lineages, it can be considered a reasonable proxy for testing hypotheses with respect to expressed niche preferences, at least with regard to the major abiotic conditions experienced by the species (Fontanella et al., 2012; Kozak et al., 2008; Warren, 2012). The ENM results thus provide support to previous findings that bank vole lineages differ in their ecological tolerances and hence performance under specific climatic conditions (Kotlík et al., 2014; Searle et al., 2009; Strážnická et al., 2018).

Our study illustrates that models built by pooling across lineages within species showing phylogeographic structure may obscure the potential that these lineages occupy distinct niches, something that suggests individual response of the intraspecific lineages to climate variation (Pearman et al., 2010). Incorporating information on intraspecific phylogeographic structure in ENM thus allows identifying not only the contribution of the intraspecific lineages to the niche occupancy of species but also the potentially distinct responses of lineages to climate change (Pahad et al., 2020). Some lineages might be better adapted to changing climate than others and gene flow, and admixture between genomes of different lineages may therefore facilitate adaptation of local populations by providing adaptive alleles (Horníková et al., 2021), which may be particularly relevant during future rapid climate change where adaptation from new mutation is likely to be less important (Hoffmann & Sgrò, 2011).

## CONFLICT OF INTEREST

None declared.

## AUTHOR CONTRIBUTION

**Marco A. Escalante:** Conceptualization (supporting); Investigation (lead); Visualization (lead); Writing‐original draft (lead); Writing‐review & editing (supporting). **Michaela Horníková:** Investigation (supporting); Writing‐review & editing (supporting). **Silvia Marková:** Investigation (supporting); Writing‐review & editing (supporting). **Petr Kotlik:** Conceptualization (lead); Investigation (supporting); Supervision (lead); Writing‐original draft (supporting); Writing‐review & editing (lead).

## Supporting information

Appendix S1

Appendix S2

## Data Availability

MaxEnt input and output data are accessible at Dryad Digital Repository (https://doi.org/10.5061/dryad.jm63xsjb0).
