# Supplementary material for: Niche differentiation in a postglacial colonizer, the bank vole Clethrionomys glareolus
Source: Ecol Evol. 2021 May 17;11(12):8054–70. doi: 10.1002/ece3.7637 (PMC8216960; doi:10.1002/ece3.7637)
Supplement: Supplementary file 1 — Appendix S1 [file ECE3-11-8054-s001.docx]

**Supporting Information**

**Niche differentiation in a postglacial colonizer, the bank vole *Clethrionomys glareolus***

Marco A. Escalante, Michaela Horníková, Silvia Marková and Petr Kotlík

**Appendix S1.** Bank vole occurrences.

| Locality | Country | Longitude | Latitude | Genetic lineage | Source |
| --- | --- | --- | --- | --- | --- |
| Bialowieza | Poland | 23.850 | 52.667 | Eastern | Wójcik et al., 2010 |
| Sobibor | Poland | 23.633 | 51.467 | Eastern | Wójcik et al., 2010 |
| Krynszczak | Poland | 22.350 | 51.983 | Eastern | Wójcik et al., 2010 |
| Lochow | Poland | 21.683 | 52.517 | Eastern | Wójcik et al., 2010 |
| Zytkiejmy | Poland | 22.667 | 54.333 | Eastern | Wójcik et al., 2010 |
| Urwitałt | Poland | 21.683 | 53.800 | Carpathian/Eastern | Wójcik et al., 2010 |
| Strzalowo | Poland | 21.450 | 53.767 | Carpathian/Eastern | Wójcik et al., 2010 |
| Zagoz dzon | Poland | 21.433 | 51.467 | Carpathian/Eastern | Wójcik et al., 2010 |
| Lipa | Poland | 22.050 | 50.683 | Carpathian/Eastern | Wójcik et al., 2010 |
| Ruda Rozaniecka | Poland | 23.183 | 50.317 | Carpathian/Eastern | Wójcik et al., 2010 |
| Wisniowa | Poland | 21.633 | 49.867 | Carpathian/Eastern/Western | Wójcik et al., 2010 |
| Cisna | Poland | 22.317 | 49.200 | Carpathian/Eastern | Wójcik et al., 2010 |
| Morskie Oko | Poland | 20.100 | 49.250 | Carpathian/Eastern | Wójcik et al., 2010 |
| Olkusz | Poland | 19.550 | 50.267 | Carpathian/Eastern | Wójcik et al., 2010 |
| Polichno | Poland | 19.700 | 50.900 | Carpathian/Eastern | Wójcik et al., 2010 |
| Przysucha | Poland | 20.633 | 51.333 | Carpathian/Western | Wójcik et al., 2010 |
| Rogalice | Poland | 17.583 | 50.950 | Carpathian | Wójcik et al., 2010 |
| Swierzawa | Poland | 15.883 | 51.000 | Carpathian/Western | Wójcik et al., 2010 |
| Wschowa | Poland | 16.300 | 51.800 | Carpathian | Wójcik et al., 2010 |
| Rzepin | Poland | 14.817 | 52.333 | Carpathian/Western | Wójcik et al., 2010 |
| Kobylnica | Poland | 17.067 | 52.433 | Carpathian | Wójcik et al., 2010 |
| Popowo Podlesne | Poland | 17.550 | 52.650 | Carpathian | Wójcik et al., 2010 |
| Lesno | Poland | 18.933 | 53.833 | Carpathian | Wójcik et al., 2010 |
| Ilawa | Poland | 19.550 | 53.583 | Carpathian | Wójcik et al., 2010 |
| Kadyny | Poland | 19.483 | 54.283 | Carpathian | Wójcik et al., 2010 |
| Stary Krakow | Poland | 16.600 | 54.433 | Carpathian | Wójcik et al., 2010 |
| Rozdoly | Poland | 14.717 | 53.283 | Carpathian | Wójcik et al., 2010 |
| Langen Brutz, Parchim | Germany | 11.550 | 53.633 | Eastern | Wójcik et al., 2010 |
| Street Dinas, Shropshire | Great Britain | -3.000 | 52.940 | Western | Searle et al., 2009 |
| Longsdon, Staffordshire | Great Britain | -2.070 | 53.100 | Western | Searle et al., 2009 |
| Longslow, Staffordshire | Great Britain | -2.510 | 52.920 | Western | Searle et al., 2009 |
| Bradley Hill, Hampshire | Great Britain | 1.080 | 51.190 | Carpathian | Searle et al., 2009 |
| Ashley, Gloucestershire | Great Britain | -2.110 | 51.650 | Western | Searle et al., 2009 |
| Ready Token, Gloucestershire | Great Britain | -1.870 | 51.740 | Western | Searle et al., 2009 |
| Ryton, Gloucestershire | Great Britain | -2.410 | 51.990 | Western | Searle et al., 2009 |
| Littleworth, Worcestershire | Great Britain | -2.020 | 52.270 | Western | Searle et al., 2009 |
| Long Marston, Warwickshire | Great Britain | -1.780 | 52.130 | Western | Searle et al., 2009 |
| Throckmorton, Worcestershire | Great Britain | -2.020 | 52.150 | Western | Searle et al., 2009 |
| Weston Jones, Staffordshire | Great Britain | -2.370 | 52.810 | Western | Searle et al., 2009 |
| Tong Forge, Shropshire | Great Britain | -2.330 | 52.670 | Western | Searle et al., 2009 |
| York | Great Britain | -1.040 | 53.940 | Western | Searle et al., 2009 |
| Gayton Thorpe | Great Britain | 0.590 | 52.740 | Western | Searle et al., 2009 |
| Grimston | Great Britain | 0.560 | 52.760 | Western | Searle et al., 2009 |
| Gazeley | Great Britain | 0.520 | 52.250 | Western | Searle et al., 2009 |
| Kentford | Great Britain | 0.520 | 52.260 | Western | Searle et al., 2009 |
| Bonnington, Bilsington | Great Britain | 0.940 | 51.070 | Carpathian | Searle et al., 2009 |
| Pevensey | Great Britain | 0.320 | 50.840 | Carpathian | Searle et al., 2009 |
| Pontyberem | Great Britain | -4.160 | 51.760 | Western | Searle et al., 2009 |
| Llannon | Great Britain | -4.150 | 51.750 | Western | Searle et al., 2009 |
| Llanddowror | Great Britain | -4.580 | 51.790 | Western | Searle et al., 2009 |
| Tavernspite, Pembrokeshire | Great Britain | -4.660 | 51.770 | Western | Searle et al., 2009 |
| Bickleigh, Devon | Great Britain | -4.070 | 50.450 | Carpathian | Searle et al., 2009 |
| Meysey Hampton Manor, Fairford, Gloucestershire | Great Britain | -1.820 | 51.720 | Western | Searle et al., 2009 |
| South Devon | Great Britain | -3.500 | 50.500 | Carpathian | Searle et al., 2009 |
| Kielder Forest, Northumberland | Great Britain | -2.590 | 55.230 | Western | Searle et al., 2009 |
| Dalgety Bay | Great Britain | -3.340 | 56.040 | Carpathian | Searle et al., 2009 |
| Linlithgow | Great Britain | -3.600 | 55.970 | Western | Searle et al., 2009 |
| Perthshire | Great Britain | -4.080 | 56.810 | Carpathian | Searle et al., 2009 |
| Oxwell | Great Britain | -2.470 | 55.980 | Western | Searle et al., 2009 |
| Thorntonloch | Great Britain | -2.390 | 55.950 | Western | Searle et al., 2009 |
| Kippford | Great Britain | -3.820 | 54.870 | Western | Searle et al., 2009 |
| Glencarse, St Madoes | Great Britain | -3.310 | 56.380 | Carpathian | Searle et al., 2009 |
| Glen Artney, Milntuirn | Great Britain | -4.000 | 56.350 | Carpathian | Searle et al., 2009 |
| Kiltarlity, Foxhole | Great Britain | -4.460 | 57.400 | Carpathian | Searle et al., 2009 |
| Avoch | Great Britain | -4.170 | 57.570 | Carpathian | Searle et al., 2009 |
| Milton of Redcastle | Great Britain | -4.370 | 57.510 | Carpathian | Searle et al., 2009 |
| Tomatin | Great Britain | -3.960 | 57.340 | Carpathian | Searle et al., 2009 |
| Torbreck | Great Britain | -4.250 | 57.440 | Carpathian | Searle et al., 2009 |
| Strathpeffer | Great Britain | -4.530 | 57.590 | Carpathian | Searle et al., 2009 |
| North Kessock | Great Britain | -4.240 | 57.500 | Carpathian | Searle et al., 2009 |
| Kirkhill | Great Britain | -4.420 | 57.450 | Carpathian | Searle et al., 2009 |
| Bunacaimb | Great Britain | -5.860 | 56.930 | Carpathian | Searle et al., 2009 |
| Bearsden | Great Britain | -4.340 | 55.920 | Western | Searle et al., 2009 |
| Medstead | Great Britain | -1.060 | 51.130 | Carpathian | Searle et al., 2009 |
| Brambridge | Great Britain | -1.330 | 51.000 | Western | Searle et al., 2009 |
| Dorset | Great Britain | -2.430 | 50.720 | Carpathian | Searle et al., 2009 |
| Frampton | Great Britain | -2.540 | 50.740 | Carpathian | Searle et al., 2009 |
| Hexham, Acomb Mill | Great Britain | -2.110 | 54.990 | Western | Searle et al., 2009 |
| Leicester area | Great Britain | -1.130 | 52.630 | Western | Searle et al., 2009 |
| Tayinloan | Great Britain | -5.670 | 55.630 | Carpathian | Searle et al., 2009 |
| Southend | Great Britain | -5.640 | 55.310 | Carpathian | Searle et al., 2009 |
| Poolewe, Ardlair | Great Britain | -5.540 | 57.730 | Carpathian | Searle et al., 2009 |
| Bridge of Earn | Great Britain | -3.410 | 56.340 | Carpathian | Searle et al., 2009 |
| Pennington | Great Britain | -3.150 | 54.200 | Western | Searle et al., 2009 |
| Epworth | Great Britain | -0.820 | 53.530 | Western | Searle et al., 2009 |
| Longtown | Great Britain | -2.970 | 55.010 | Western | Searle et al., 2009 |
| Brampton, Banks | Great Britain | -2.670 | 54.970 | Western | Searle et al., 2009 |
| Invergordon, Tullich, Druminault | Great Britain | -4.150 | 57.750 | Carpathian | Searle et al., 2009 |
| Hopwood | Great Britain | -1.960 | 52.370 | Western | Searle et al., 2009 |
| Ambergate | Great Britain | -1.490 | 53.060 | Western | Searle et al., 2009 |
| Whitstable | Great Britain | 1.020 | 51.350 | Carpathian | Searle et al., 2009 |
| Bromley | Great Britain | 0.080 | 51.350 | Carpathian | Searle et al., 2009 |
| Whitstable, Pean Hill | Great Britain | 1.020 | 51.330 | Carpathian | Searle et al., 2009 |
| Herne Common | Great Britain | 1.110 | 51.330 | Carpathian | Searle et al., 2009 |
| Ashbourne, Kirk Langley | Great Britain | -1.570 | 52.940 | Western | Searle et al., 2009 |
| Corsham | Great Britain | -2.190 | 51.440 | Western | Searle et al., 2009 |
| Mildenhall | Great Britain | 0.530 | 52.340 | Western | Searle et al., 2009 |
| Clarach | Great Britain | -4.070 | 52.430 | Western | Searle et al., 2009 |
| Capel Mawr, Paradwys | Great Britain | -4.360 | 53.210 | Western | Searle et al., 2009 |
| Cartmel, Aynsome | Great Britain | -2.950 | 54.200 | Western | Searle et al., 2009 |
| Cowal | Great Britain | -5.080 | 55.900 | Carpathian | Searle et al., 2009 |
| Balmacara | Great Britain | -5.640 | 57.300 | Carpathian | Searle et al., 2009 |
| Craignure | Great Britain | -5.700 | 56.460 | Carpathian | Searle et al., 2009 |
| Brodick | Great Britain | -5.150 | 55.600 | Carpathian | Searle et al., 2009 |
| Tobermory | Great Britain | -6.100 | 56.620 | Carpathian | Searle et al., 2009 |
| Monk's Heath | Great Britain | -2.230 | 53.250 | Western | Searle et al., 2009 |
| Pusey | Great Britain | -1.470 | 51.670 | Western | Searle et al., 2009 |
| Loch Lomond | Great Britain | -4.600 | 56.070 | Western | Searle et al., 2009 |
| Pickering, Dalby Forest, Ellerburn Road | Great Britain | -0.694 | 54.254 | Western | Marková et al., 2020 |
| Pickering, Dalby Forest, Dalby Forest Drive | Great Britain | -0.680 | 54.292 | Western | Marková et al., 2020 |
| Heslington | Great Britain | -1.010 | 53.928 | Western | Marková et al., 2020 |
| Doncaster, Loversall | Great Britain | -1.133 | 53.489 | Western | Kotlík et al., 2014 |
| Doncaster, Wadworth | Great Britain | -1.134 | 53.473 | Western | Kotlik et al., 2014 |
| Roslin, Carrington | Great Britain | -3.102 | 55.836 | Western | Marková et al., 2020 |
| Roslin, Carrington | Great Britain | -3.103 | 55.847 | Western | Marková et al., 2020 |
| Maud, Annochie | Great Britain | -2.081 | 57.480 | Carpathian | Marková et al., 2020 |
| Isle of Raasay, Inverarish | Great Britain | -6.068 | 57.357 | Carpathian | Marková et al., 2020 |
| Isle of Raasay, Brochel | Great Britain | -6.037 | 57.441 | Carpathian | Searle et al., 2009 |
| Isle of Mull, Salen, Kellan | Great Britain | -6.015 | 56.499 | Carpathian | Marková et al., 2020 |
| Isle of Mull, Salen, Gruline | Great Britain | -5.991 | 56.486 | Carpathian | Marková et al., 2020 |
| Wicken, Headlake Drove | Great Britain | 0.267 | 52.279 | Western | Marková et al., 2020 |
| Wicken, Upware Road | Great Britain | 0.265 | 52.315 | Western | Marková et al., 2020 |
| Diptford, Elwell | Great Britain | -3.785 | 50.413 | Carpathian | Kotlík et al., 2006 |
| Diptford, West Leigh | Great Britain | -3.754 | 50.411 | Carpathian | Kotlik et al., 2006 |
| Diptford, East Moore | Great Britain | -3.763 | 50.413 | Carpathian | Kotlik et al., 2006 |
| Little Thurlow | Great Britain | 0.439 | 52.129 | Western | Marková et al., 2020 |
| Temple End | Great Britain | 0.410 | 52.135 | Carpathian | Marková et al., 2020 |
| Westley Bottom Road | Great Britain | 0.330 | 52.194 | Western | Marková et al., 2020 |
| Westley Waterless | Great Britain | 0.345 | 52.168 | Carpathian | Marková et al., 2020 |
| North Hill | Great Britain | -4.447 | 50.558 | Carpathian | Marková et al., 2020 |
| Trebartha | Great Britain | -4.438 | 50.573 | Carpathian | Marková et al., 2020 |
| Cury, Lizard Peninsula | Great Britain | -5.247 | 50.051 | Carpathian | Marková et al., 2020 |
| Zoar, St Keverne, Lizard Peninsula | Great Britain | -5.128 | 50.043 | Carpathian | Marková et al., 2020 |
| Winson, Cirencester | Great Britain | -1.889 | 51.770 | Western | Marková et al., 2020 |
| Westwell, Burford, Cirencester | Great Britain | -1.695 | 51.778 | Western | Marková et al., 2020 |
| Duntisbourne Abbots, Cirencester | Great Britain | -2.054 | 51.766 | Western | Marková et al., 2020 |
| Duntisbourne Rouse, Longhill Road, Cirencester | Great Britain | -2.033 | 51.750 | Western | Marková et al., 2020 |
| Feofania Forest, Kiev | Ukraine | 30.470 | 50.330 | Eastern | Kotlik et al., 2006 |
| Rila Mts | Bulgaria | 23.400 | 42.000 | Southern | Kotlik et al., 2006 |
| Southern Mts | Bulgaria | 23.400 | 43.100 | Southern | Kotlik et al., 2006 |
| W Rhodopi Mts | Bulgaria | 24.500 | 41.500 | Southern | Kotlik et al., 2006 |
| Vitosha Mts | Bulgaria | 23.000 | 42.200 | Southern | Kotlik et al., 2006 |
| Britavka | Ukraine | 29.130 | 48.170 | Eastern | Kotlik et al., 2006 |
| Rovno | Ukraine | 25.950 | 50.800 | Eastern | Kotlik et al., 2006 |
| Nagycsany | Hungary | 17.950 | 45.870 | Western | Kotlik et al., 2006 |
| Solnik, Varna | Bulgaria | 27.650 | 42.930 | Southern | Deffontaine et al., 2005 |
| Elena, Veliko Tarnovo | Bulgaria | 25.860 | 42.880 | Southern | Kotlik et al., 2006 |
| Bacau | Romania | 26.830 | 46.550 | Carpathian/Eastern/Western | Kotlik et al., 2006 |
| Bohdašín, Náchod | Czech Republic | 16.120 | 50.500 | Carpathian/Western | Kotlik et al., 2006 |
| Praha Kunratice | Czech Republic | 14.480 | 50.020 | Carpathian/Western | Kotlik et al., 2006 |
| Uhersky Brod, Nezdenice | Czech Republic | 17.760 | 49.010 | Carpathian | Kotlik et al., 2006 |
| Jezersko, Kezmarok | Slovakia | 20.350 | 49.300 | Carpathian | Kotlik et al., 2006 |
| Remetske Hamre | Slovakia | 22.180 | 48.840 | Eastern | Kotlik et al., 2006 |
| Domaniky, Krupina | Slovakia | 18.990 | 48.260 | Carpathian | Kotlik et al., 2006 |
| Oravice, Sucha dolina | Slovakia | 19.750 | 49.200 | Carpathian | Kotlik et al., 2006 |
| Biogradsko Jezero | Montenegro | 19.610 | 42.890 | Western | Marková et al., 2020 |
| Mušov-Betlém | Czech Republic | 16.600 | 48.920 | Carpathian | Marková et al., 2020 |
| Kašperské Hory, Klatovy | Czech Republic | 13.570 | 49.100 | Western | Kotlik et al., 2006 |
| Taga Cluj, Transylvania | Romania | 24.050 | 46.950 | Carpathian | Marková et al., 2020 |
| Rozhanovce, Košice | Slovakia | 21.370 | 48.770 | Carpathian/Eastern | Kotlik et al., 2006 |
| Filipov, Děčín | Czech Republic | 14.380 | 50.820 | Carpathian/Western | Kotlik et al., 2006 |
| Nový Hamr, Domažlice | Czech Republic | 12.850 | 49.430 | Western | Kotlik et al., 2006 |
| Mšec | Czech Republic | 13.900 | 50.210 | Carpathian/Western | Kotlik et al., 2006 |
| Smilkov, Votice | Czech Republic | 14.620 | 49.610 | Carpathian/Western | Kotlik et al., 2006 |
| Frýdek Místek | Czech Republic | 18.380 | 49.700 | Carpathian | Kotlik et al., 2006 |
| Ostrava | Czech Republic | 18.220 | 49.800 | Carpathian | Kotlik et al., 2006 |
| Bratislava | Slovakia | 17.230 | 48.230 | Carpathian/Western | Kotlik et al., 2006 |
| Vlkov, Veselí nad Lužnicí | Czech Republic | 14.690 | 49.150 | Western | Kotlik et al., 2006 |
| České Budejovice | Czech Republic | 14.430 | 48.980 | Carpathian | Kotlik et al., 2006 |
| České Budejovice | Czech Republic | 14.510 | 49.050 | Carpathian/Western | Marková et al., 2020 |
| Portumna | Ireland | -8.230 | 53.080 | Western | Marková et al., 2020 |
| Silvermines | Ireland | -8.230 | 52.750 | Western | Marková et al., 2020 |
| Tooreenbrien Woods | Ireland | -8.270 | 52.680 | Western | Marková et al., 2020 |
| Castlelough | Ireland | -8.400 | 52.850 | Western | Marková et al., 2020 |
| Curragh Chase | Ireland | -8.850 | 52.580 | Western | Marková et al., 2020 |
| Killarney Conifer | Ireland | -9.480 | 52.020 | Western | Marková et al., 2020 |
| Kinvarra | Ireland | -8.970 | 53.130 | Western | Marková et al., 2020 |
| Strmosten | Serbia and Montenegro | 21.640 | 44.090 | Carpathian | Kotlik et al., 2006 |
| Stražilovo, Sremski Karlovci | Serbia and Montenegro | 19.910 | 45.170 | Western | Kotlik et al., 2006 |
| Kopaonik National Park | Serbia and Montenegro | 20.820 | 43.240 | Southern/Carpathian/Western | Kotlik et al., 2006 |
| Tara National Park | Serbia and Montenegro | 19.430 | 43.920 | Carpathian/Western | Kotlik et al., 2006 |
| Prachovské sedlo | Czech Republic | 15.300 | 50.470 | Carpathian | Kotlik et al., 2006 |
| Fričkovice, Bardejov | Slovakia | 21.250 | 49.180 | Carpathian/Eastern | Kotlik et al., 2006 |
| Vranovice | Czech Republic | 16.600 | 48.970 | Carpathian/Western | Kotlik et al., 2006 |
| Lednice | Czech Republic | 16.800 | 48.800 | Carpathian/Western | Kotlik et al., 2006 |
| Iadăra, Maramures | Romania | 23.400 | 47.480 | Carpathian | Kotlik et al., 2006 |
| La Stanuleti | Romania | 23.830 | 45.300 | Carpathian | Kotlik et al., 2006 |
| Feleacu, Cluj-Napoca | Romania | 23.620 | 46.720 | Eastern | Kotlik et al., 2006 |
| Kubelhof, Kulmbach | Germany | 11.380 | 50.170 | Western | Kotlik et al., 2006 |
| Kamnica & Vinje | Slovenia | 14.670 | 46.110 | Carpathian/Western | Kotlik et al., 2006 |
| Jastrebarsko | Croatia | 15.650 | 45.740 | Southern/Western | Deffontaine et al., 2005 |
| Vrbovsko | Croatia | 15.080 | 45.370 | Western | Kotlik et al., 2006 |
| Radinje, Siče | Croatia | 17.600 | 45.140 | Western | Kotlik et al., 2006 |
| Batrina, Nova Gradiška | Croatia | 17.680 | 45.200 | Western | Marková et al., 2020 |
| Radenci | Slovenia | 16.040 | 46.650 | Western | Kotlik et al., 2006 |
| Lunz am See | Austria | 15.050 | 47.850 | Western | Kotlik et al., 2006 |
| Bezau, Vorarlberg | Austria | 9.970 | 47.370 | Western | Kotlik et al., 2006 |
| Stockerau | Austria | 16.230 | 48.370 | Western/Carpathian | Kotlik et al., 2006 |
| Radurschltal, Pfunds | Austria | 10.600 | 46.920 | Western | Kotlik et al., 2006 |
| Ardez, Aargau | Switzerland | 10.180 | 46.780 | Western | Kotlik et al., 2006 |
| Cudrefin | Switzerland | 7.020 | 46.950 | Western | Kotlik et al., 2006 |
| Ócsa, Budapest | Hungary | 19.220 | 47.300 | Western | Kotlik et al., 2006 |
| Bursa | Turkey | 29.170 | 40.120 | Southern | Strážnická et al., 2018 |
| Ingolstadt | Germany | 11.430 | 48.770 | Western | Marková et al., 2020 |
| Kechnec, Košice | Slovakia | 21.270 | 48.550 | Carpathian | Marková et al., 2020 |
| Hakel forest, Quedlinburg | Germany | 11.150 | 51.780 | Western | Strážnická et al., 2018 |
| Crna rijeka, Plitvički Ljeskovac | Croatia | 15.620 | 44.830 | Western | Marková et al., 2020 |
| Rječica, Jezerce | Croatia | 15.620 | 44.850 | Western | Marková et al., 2020 |
| Plitvice Lake | Croatia | 15.620 | 44.840 | Western | Marková et al., 2020 |
| Sušanjski potok, Plitvički Ljeskovac | Croatia | 15.590 | 44.850 | Western | Marková et al., 2020 |
| Polichno | Poland | 19.820 | 51.470 | Carpathian | Strážnická et al., 2018 |
| Bialowieza National Park | Poland | 23.850 | 52.730 | Eastern | Strážnická et al., 2018 |
| Ostashkhov, Seliger Lake | Russia | 33.100 | 57.150 | Eastern | Strážnická et al., 2018 |
| Vimoutiers | France | 0.200 | 48.920 | Western | Marková et al., 2020 |
| Saint-Setiers | France | 2.130 | 45.700 | Western | Marková et al., 2020 |
| Geneva | Switzerland | 6.170 | 46.200 | Western | Strážnická et al., 2018 |
| Zurich | Switzerland | 8.550 | 47.370 | Western | Marková et al., 2020 |
| Heidenheim | Germany | 10.730 | 49.020 | Western | Marková et al., 2020 |
| Ravensburg | Germany | 9.650 | 47.810 | Western | Marková et al., 2020 |
| Ardennes | Belgium | 4.180 | 50.630 | Western | Marková et al., 2020 |
| Egholm | Denmark | 11.920 | 55.730 | Eastern | Marková et al., 2020 |
| Morges | Switzerland | 6.500 | 46.520 | Western | Marková et al., 2020 |
| Bourg St Bernard | Switzerland | 7.200 | 45.900 | Western | Marková et al., 2020 |
| Bussiéres, La Cote | France | 2.650 | 46.040 | Western | Marková et al., 2020 |
| Le Quartier, Crepaillat | France | 2.730 | 46.140 | Western | Marková et al., 2020 |
| Dontreix, Villemaloux | France | 2.550 | 46.000 | Western | Marková et al., 2020 |
| Marcillat, Outre | France | 2.630 | 46.170 | Western | Marková et al., 2020 |
| Belle Isle en Terre | France | 3.400 | 48.550 | Western | Marková et al., 2020 |
| Luxemburg | Luxemburg | 6.130 | 49.610 | Western | Marková et al., 2020 |
| Urwitalt, Luknajno | Poland | 21.650 | 53.800 | Carpathian/Eastern | Deffontaine et al., 2005 |
| Kobylnica, Poznan | Poland | 17.100 | 52.450 | Carpathian | Marková et al., 2020 |
| Uhorná | Slovakia | 20.690 | 48.700 | Carpathian/Eastern | Marková et al., 2020 |
| Krásnohorské Podhradie | Slovakia | 20.610 | 48.660 | Carpathian | Marková et al., 2020 |
| Hanišberg, Krupina | Slovakia | 19.090 | 48.396 | Carpathian/Western | Strážnická et al., 2018 |
| Belle Ile en Mer | France | -3.170 | 47.340 | Western | Marková et al., 2020 |
| High Tatra | Slovakia | 20.160 | 49.130 | Carpathian | Kotlik et al., 2006 |
| High Tatra | Slovakia | 20.160 | 49.150 | Carpathian/Western | Kotlik et al., 2006 |
| High Tatra | Slovakia | 20.080 | 49.160 | Carpathian/Western | Kotlik et al., 2006 |
| High Tatra | Slovakia | 19.980 | 49.170 | Carpathian/Eastern | Kotlik et al., 2006 |
| Esterndorf | Germany | 12.100 | 48.260 | Carpathian | Marková et al., 2020 |
| Letzlingen, Gardelegen | Germany | 11.460 | 52.500 | Western | Marková et al., 2020 |
| Varde, Ribe | Denmark | 8.480 | 55.630 | Eastern | Kotlik et al., 2006 |
| Arnum, Enderupskov | Denmark | 8.940 | 55.270 | Eastern | Marková et al., 2020 |
| Vester Vedsted | Denmark | 8.690 | 55.290 | Eastern | Marková et al., 2020 |
| Neuborn, Kropp | Germany | 9.380 | 54.410 | Eastern/Western | Marková et al., 2020 |
| Schrevendorf | Germany | 10.270 | 54.370 | Western | Marková et al., 2020 |
| Eutin | Germany | 10.620 | 54.130 | Eastern | Marková et al., 2020 |
| Weddingsteadt | Germany | 9.110 | 54.220 | Eastern | Marková et al., 2020 |
| Ostrohe | Germany | 9.110 | 54.230 | Eastern | Marková et al., 2020 |
| Oxstedt | Germany | 8.590 | 53.800 | Western | Marková et al., 2020 |
| Oxstedt | Germany | 8.580 | 53.800 | Western | Marková et al., 2020 |
| Hage, Niedersachsen | Germany | 7.270 | 53.620 | Eastern | Marková et al., 2020 |
| Ratzerburg, Moeln | Germany | 10.750 | 53.680 | Eastern/Western | Strážnická et al., 2018 |
| Dahme | Germany | 11.070 | 54.210 | Eastern/Western | Marková et al., 2020 |
| Havelberg | Germany | 12.100 | 52.840 | Eastern/Western | Marková et al., 2020 |
| Linda | Germany | 13.060 | 51.860 | Carpathian/Western | Marková et al., 2020 |
| Sila | Italy | 16.490 | 39.350 | Southern | Colangelo et al., 2011 |
| Fressennville | France | 1.570 | 50.080 | Western | Marková et al., 2020 |
| Ribeaville | France | 1.630 | 50.170 | Western | Strážnická et al., 2018 |
| Pihen lé Guines | France | 1.820 | 50.870 | Western | Marková et al., 2020 |
| Carolles | France | 1.550 | 48.750 | Western | Marková et al., 2020 |
| St Jean le Thomas | France | 1.520 | 48.720 | Western | Marková et al., 2020 |
| St. Omer | France | 2.250 | 50.750 | Western | Marková et al., 2020 |
| Guines | France | 1.870 | 50.830 | Western | Marková et al., 2020 |
| Popova Šapka | Macedonia | 20.890 | 42.020 | Southern/Western | Marková et al., 2020 |
| Ponikve | Macedonia | 22.500 | 42.167 | Southern/Carpathian | Marková et al., 2020 |
| Begova Cešma | Macedonia | 21.180 | 41.030 | Southern | Marková et al., 2020 |
| Tallinn | Estonia | 24.730 | 59.430 | Carpathian | Marková et al., 2020 |
| Le Souillot | France | 6.030 | 47.020 | Western | Marková et al., 2020 |
| Pyrenees | France | -0.120 | 43.250 | Southern | Strážnická et al., 2018 |
| Heuckewalde | Germany | 12.139 | 50.969 | Western | Marková et al., 2020 |
| Breitenbach | Germany | 12.076 | 50.994 | Western | Marková et al., 2020 |
| Duelsberg | Germany | 10.833 | 52.795 | Eastern/Western | Marková et al., 2020 |
| Diesdorf | Germany | 10.880 | 52.766 | Eastern | Marková et al., 2020 |
| Želízy | Czech Republic | 14.470 | 50.420 | Carpathian/Western | Marková et al., 2020 |
| Dieren, Arnhem | Netherlands | 6.076 | 52.071 | Western | Marková et al., 2020 |
| Bakkeveen | Netherlands | 6.244 | 53.072 | Western | Marková et al., 2020 |
| Callantsoog | Netherlands | 4.698 | 52.804 | Western | Marková et al., 2020 |
| Noordwijk | Netherlands | 4.470 | 52.248 | Western | Strážnická et al., 2018 |
| Skog | Sweden | 16.808 | 61.145 | Carpathian | Strážnická et al., 2018 |
| Bollnas | Sweden | 16.285 | 61.453 | Carpathian | Strážnická et al., 2018 |
| Kramfors | Sweden | 17.723 | 62.879 | Carpathian/Rutilus | Strážnická et al., 2018 |
| Kramfors | Sweden | 17.746 | 62.880 | Carpathian/Rutilus | Strážnická et al., 2018 |
| Buckarby | Sweden | 16.980 | 60.217 | Carpathian | Strážnická et al., 2018 |
| Harbo | Sweden | 17.317 | 60.091 | Carpathian | Strážnická et al., 2018 |
| Arla | Sweden | 16.789 | 59.295 | Carpathian | Strážnická et al., 2018 |
| Arla | Sweden | 16.702 | 59.294 | Carpathian | Strážnická et al., 2018 |
| Valla | Sweden | 16.357 | 58.995 | Carpathian | Strážnická et al., 2018 |
| Stensoffa | Sweden | 13.488 | 55.711 | Carpathian | Strážnická et al., 2018 |
| Stensoffa | Sweden | 13.462 | 55.688 | Carpathian | Strážnická et al., 2018 |
| Torsby | Sweden | 12.811 | 60.141 | Carpathian | Strážnická et al., 2018 |
| Torsby | Sweden | 12.763 | 60.169 | Carpathian | Strážnická et al., 2018 |
| Torsby | Sweden | 12.745 | 60.194 | Carpathian | Strážnická et al., 2018 |
| Lolland, Guldborg | Denmark | 11.710 | 54.872 | Eastern | Marková et al., 2020 |
| Lolland, Killerup | Denmark | 11.667 | 54.828 | Eastern | Marková et al., 2020 |
| Lolland, Tjennemarke | Denmark | 11.365 | 54.808 | Eastern | Marková et al., 2020 |
| Strandkaer, Molslaboratoriet | Denmark | 10.569 | 56.227 | Eastern | Marková et al., 2020 |
| Calabria, Catena Costiera | Italy | 16.116 | 39.301 | Southern | Strážnická et al., 2018 |
| Apulia | Italy | 16.009 | 41.817 | Southern | Deffontaine et al., 2005 |
| Snasa | Norway | 12.049 | 64.171 | Carpathian | Strážnická et al., 2018 |
| Valay, Steinkjer | Norway | 11.931 | 64.139 | Carpathian | Marková et al., 2020 |
| Surnadal | Norway | 8.714 | 62.963 | Carpathian | Marková et al., 2020 |
| Venabygd, Ringebu | Norway | 10.062 | 61.583 | Carpathian | Kotlik et al., 2006 |
| Eksingedal, Vaksdal | Norway | 5.974 | 60.790 | Carpathian | Strážnická et al., 2018 |
| Ose, Bygland | Norway | 7.657 | 58.979 | Carpathian | Strážnická et al., 2018 |
| Lillesand | Norway | 8.227 | 58.243 | Carpathian | Kotlik et al., 2006 |
| Sandeled, Risar | Norway | 9.212 | 58.767 | Carpathian | Marková et al., 2020 |
| Harestua, Oppland | Norway | 10.726 | 60.222 | Carpathian | Marková et al., 2020 |
| Göteborg, Hällingsjö | Sweden | 12.419 | 57.621 | Carpathian | Strážnická et al., 2018 |
| Viterbo | Italy | 12.120 | 42.517 | Southern | Deffontaine et al., 2005 |
| La Selva Forest | Italy | 11.067 | 43.217 | Southern | Deffontaine et al., 2005 |
| Armendarits | France | 1.173 | 43.302 | Southern | Deffontaine et al., 2009 |
| Foreste Casentinesi National Park | Italy | 11.838 | 43.831 | Southern | Marková et al., 2020 |
| Abetina Di Rosello Regional Natural Reserve | Italy | 14.352 | 41.887 | Southern | Marková et al., 2020 |
| Gfrill | Italy | 11.125 | 46.536 | Western | Marková et al., 2020 |
| Korpilahti | Finland | 25.570 | 61.990 | Eastern | Marková et al., 2020 |
| Kuhmoinen | Finland | 25.200 | 61.560 | Eastern | Marková et al., 2020 |
| Luhanka | Finland | 25.710 | 61.800 | Eastern | Marková et al., 2020 |
| Lieksa, Koli | Finland | 29.808 | 63.108 | Eastern | Marková et al., 2020 |
| Suonenjoki | Finland | 27.103 | 62.634 | Eastern | Marková et al., 2020 |
| Virolahti | Finland | 27.680 | 60.590 | Eastern | Marková et al., 2020 |
| Luumäki | Finland | 27.595 | 60.917 | Eastern | Marková et al., 2020 |
| Tohmajärvi | Finland | 30.360 | 62.225 | Eastern | Marková et al., 2020 |
| Mikkeli | Finland | 27.100 | 61.800 | Eastern | Marková et al., 2020 |
| Vammala | Finland | 22.890 | 61.370 | Eastern | Marková et al., 2020 |
| Bucharest | Romania | 26.170 | 44.420 | Eastern | Kotlik et al., 2006 |
| Satic, Judetul Arges | Romania | 25.170 | 45.380 | Eastern | Marková et al., 2020 |
| Pravenec, Prievidza | Slovakia | 18.660 | 48.840 | Carpathian/Western | Marková et al., 2020 |
| Chernobyl | Ukraine | 29.914 | 51.185 | Eastern | Marková et al., 2020 |
| Chernobyl | Ukraine | 29.969 | 51.163 | Eastern | Marková et al., 2020 |
| Chernobyl | Ukraine | 30.211 | 51.212 | Eastern | Marková et al., 2020 |
| Chernobyl | Ukraine | 30.144 | 51.204 | Eastern | Marková et al., 2020 |
| Chernobyl | Ukraine | 29.964 | 51.149 | Eastern | Marková et al., 2020 |
| Chernobyl | Ukraine | 30.193 | 51.213 | Eastern | Marková et al., 2020 |
| Chernobyl | Ukraine | 30.004 | 51.163 | Eastern | Marková et al., 2020 |
| Chernobyl | Ukraine | 29.938 | 51.139 | Eastern | Marková et al., 2020 |
| Chernobyl | Ukraine | 30.122 | 51.361 | Eastern | Marková et al., 2020 |
| Chernobyl | Ukraine | 30.109 | 51.206 | Eastern | Marková et al., 2020 |
| Chernobyl | Ukraine | 30.032 | 51.371 | Eastern | Marková et al., 2020 |
| Chernobyl | Ukraine | 29.992 | 51.380 | Eastern | Marková et al., 2020 |
| Rila | Bulgaria | 23.361 | 42.042 | Southern | Kotlik et al., 2006 |
| Rila | Bulgaria | 23.384 | 42.041 | Southern | Kotlik et al., 2006 |
| Vitosha | Bulgaria | 23.253 | 42.601 | Southern | Kotlik et al., 2006 |
| Târgu Mureş | Romania | 20.040 | 46.350 | Eastern | Deffontaine et al., 2009 |
| Asturias | Spain | -6.400 | 43.000 | Southern | Deffontaine et al., 2009 |
| Colonster | Belgium | 5.330 | 50.390 | Western | Deffontaine et al., 2009 |
| Dalhem | Belgium | 5.430 | 50.420 | Western | Deffontaine et al., 2009 |
| Blegny Trembleur | Belgium | 5.440 | 50.400 | Western | Deffontaine et al., 2009 |
| Virelles | Belgium | 4.200 | 50.030 | Western | Deffontaine et al., 2005 |
| Leernes | Belgium | 4.190 | 50.230 | Western | Deffontaine et al., 2009 |
| Ginzling | Austria | 11.490 | 47.050 | Western | Deffontaine et al., 2009 |
| Kärnten | Austria | 12.819 | 47.068 | Western | Deffontaine et al., 2005 |
| Osttirol | Austria | 12.230 | 49.923 | Western | Deffontaine et al., 2005 |
| Osttirol | Austria | 12.217 | 46.894 | Western | Deffontaine et al., 2005 |
| Salzburg | Austria | 13.080 | 47.370 | Western | Deffontaine et al., 2005 |
| Dordogne,Bourdeilles | France | 0.566 | 45.323 | Western | Deffontaine et al., 2005 |
| Dordogne,Paussac | France | 0.528 | 45.328 | Western | Deffontaine et al., 2009 |
| Oriental Pyrenees | France | 2.190 | 42.290 | Western | Deffontaine et al., 2009 |
| Creuse, Saint Merd les Oussines | France | 2.029 | 45.631 | Southernn | Deffontaine et al., 2009 |
| Arolla | Switzerland | 7.280 | 46.010 | Western | Deffontaine et al., 2009 |
| Arolla | Switzerland | 7.290 | 47.070 | Western | Deffontaine et al., 2009 |
| Essex | Great Britain | 0.570 | 51.770 | Western | Deffontaine et al., 2005 |
| Bakewell, Derbyshire | Great Britain | -1.675 | 53.197 | Western | Deffontaine et al., 2005 |
| High cross, Cambridgeshire | Great Britain | 0.084 | 52.362 | Western | Deffontaine et al., 2005 |
| Gwent | Great Britain | -3.200 | 51.761 | Western | Deffontaine et al., 2005 |
| Kielder | Great Britain | -2.340 | 55.130 | Western | Deffontaine et al., 2005 |
| Zehra | Slovakia | 20.800 | 48.980 | Carpathian | Deffontaine et al., 2009 |
| Maramures, Baia Mare | Romania | 23.550 | 47.550 | Eastern | Deffontaine et al., 2005 |
| Crisana, Ineu | Romania | 22.140 | 46.270 | Carpathian/Eastern | Deffontaine et al., 2009 |
| Banat, Baile Herculane | Romania | 22.250 | 44.520 | Southern/Eastern | Deffontaine et al., 2005 |
| Zarnesti, Plaiul Foii | Romania | 26.520 | 47.170 | Eastern | Deffontaine et al., 2005 |
| Bacau, Coman | Romania | 26.480 | 46.280 | Western | Deffontaine et al., 2005 |
| Livek | Slovenia | 13.340 | 46.120 | Carpathian | Deffontaine et al., 2005 |
| Delnice | Slovenia | 14.110 | 46.090 | Western | Deffontaine et al., 2009 |
| Rut | Slovenia | 13.530 | 46.110 | Western | Deffontaine et al., 2009 |
| Zala, Bak | Hungary | 16.500 | 46.430 | Western | Deffontaine et al., 2005 |
| Baranya, Pecs | Hungary | 18.130 | 46.040 | Western | Deffontaine et al., 2009 |
| Gera, Langenberg | Germany | 12.050 | 50.520 | Western | Deffontaine et al., 2005 |
| Langen Brulz | Germany | 12.190 | 53.320 | Eastern | Deffontaine et al., 2009 |
| Pulawy | Poland | 21.580 | 51.250 | Eastern | Deffontaine et al., 2005 |
| Cuneo,Pietraporzio | Italy | 7.028 | 44.331 | Southern | Deffontaine et al., 2005 |
| Cap d'Acqua | Italy | 12.540 | 42.040 | Southern | Deffontaine et al., 2009 |
| Toscane, Chiusi della Verna | Italy | 11.932 | 43.698 | Southern | Deffontaine et al., 2005 |
| Mont Pelister | Macedonia | 21.227 | 40.982 | Southern | Deffontaine et al., 2005 |
| National Park Zemaitijos | Lituania | 21.560 | 56.050 | Carpathian/Eastern | Deffontaine et al., 2005 |
| Alytus | Lituania | 24.020 | 54.230 | Eastern | Deffontaine et al., 2005 |
| Navarra | Spain | -1.300 | 42.590 | Southern | Deffontaine et al., 2005 |
| Horna | Spain | -3.340 | 42.560 | Southern | Deffontaine et al., 2009 |
| Uludag mountains | Turkey | 29.185 | 40.126 | Southern | Deffontaine et al., 2005 |
| Pohorje Massiv | Slovenia | 15.300 | 46.320 | Western | Deffontaine et al., 2009 |
| Goteniska gora, Kocevje | Slovenia | 14.510 | 45.380 | Western | Deffontaine et al., 2009 |
| Ljubljana | Slovenia | 14.300 | 46.040 | Western | Deffontaine et al., 2009 |
| Glazuta, Ribnica | Slovenia | 14.420 | 45.460 | Western | Deffontaine et al., 2009 |
| Orehek, Kozina | Slovenia | 13.560 | 45.360 | Western | Deffontaine et al., 2009 |
| Delnice | Croatia | 15.050 | 45.250 | Western | Deffontaine et al., 2009 |
| Delnice | Croatia | 15.030 | 45.260 | Western | Deffontaine et al., 2009 |
| Gracac SE | Croatia | 16.050 | 44.170 | Western | Deffontaine et al., 2009 |
| Bourdeilles | France | 0.590 | 45.320 | Western | Deffontaine et al., 2009 |
| Pausac | France | 0.310 | 45.210 | Western | Deffontaine et al., 2009 |
| Cadouin | France | 0.520 | 44.480 | Western | Deffontaine et al., 2009 |
| Néouvielle | France | 0.070 | 42.560 | Southern | Deffontaine et al., 2009 |
| Py Mantent | France | 2.350 | 42.500 | Southern | Deffontaine et al., 2009 |
| Saint Aignan | France | 2.320 | 47.850 | Western | Deffontaine et al., 2009 |
| Navarre | Spain | -1.500 | 42.980 | Southern | Deffontaine et al., 2009 |
| Chadroma | Russia | 42.933 | 61.200 | Eastern | Abramson et al., 2009 |
| Koslan | Russia | 48.733 | 63.433 | Eastern | Abramson et al., 2009 |
| Belgorod oblast | Russia | 36.583 | 50.600 | Eastern | Abramson et al., 2009 |
| Vladimir oblast | Russia | 40.183 | 55.517 | Eastern | Abramson et al., 2009 |
| Kaliningrad oblast | Russia | 20.833 | 55.150 | Eastern | Abramson et al., 2009 |
| Pitkyaranta raion, near village of Karkku | Russia | 31.933 | 61.283 | Eastern | Abramson et al., 2009 |
| Kondopoga | Russia | 34.317 | 62.183 | Eastern | Abramson et al., 2009 |
| Dan’ | Russia | 51.800 | 61.383 | Eastern | Abramson et al., 2009 |
| Priozernyi | Russia | 51.817 | 61.783 | Eastern | Abramson et al., 2009 |
| Storozhevsk | Russia | 52.333 | 61.950 | Eastern | Abramson et al., 2009 |
| Pechoro-Ilychsky National Reserve | Russia | 58.433 | 62.100 | Eastern | Abramson et al., 2009 |
| Village of Shul’gino | Russia | 34.783 | 59.217 | Eastern | Abramson et al., 2009 |
| Malyi Berezovyi Island | Russia | 28.483 | 60.317 | Eastern | Abramson et al., 2009 |
| Village of Kurgolovo | Russia | 28.100 | 59.767 | Eastern | Abramson et al., 2009 |
| Village of Zhelezo | Russia | 29.850 | 58.733 | Eastern | Abramson et al., 2009 |
| Villages of Izvara, Milodezh’, and Chernoe | Russia | 29.483 | 59.317 | Eastern | Abramson et al., 2009 |
| Tikhvin raion, village of Zabor’e | Russia | 34.950 | 60.117 | Eastern | Abramson et al., 2009 |
| Valaam Island | Russia | 30.950 | 61.367 | Eastern | Abramson et al., 2009 |
| Village of Zaostrov’e | Russia | 33.550 | 60.717 | Eastern | Abramson et al., 2009 |
| Pulkovo | Russia | 30.317 | 59.783 | Eastern | Abramson et al., 2009 |
| Vologda oblast | Russia | 35.800 | 59.433 | Eastern | Abramson et al., 2009 |
| Novgorod oblast | Russia | 31.250 | 58.533 | Eastern | Abramson et al., 2009 |
| Orenburg oblast | Russia | 55.000 | 51.533 | Eastern | Abramson et al., 2009 |
| Pskov oblast | Russia | 29.117 | 58.267 | Eastern | Abramson et al., 2009 |
| Pskov oblast | Russia | 27.933 | 56.033 | Eastern | Abramson et al., 2009 |
| Saratov oblast | Russia | 44.367 | 52.050 | Eastern | Abramson et al., 2009 |
| Sverdlovsk oblast | Russia | 59.583 | 56.667 | Eastern | Abramson et al., 2009 |
| villages of Khomutovka and Shigarevo, Visim National Park, and Yekaterinburg | Russia | 59.750 | 57.367 | Eastern | Abramson et al., 2009 |
| Tver oblast | Russia | 35.050 | 57.317 | Eastern | Abramson et al., 2009 |
| Moscow oblast | Russia | 36.883 | 55.717 | Eastern | Abramson et al., 2009 |
| Tomsk oblast | Russia | 77.183 | 60.483 | Eastern | Abramson et al., 2009 |
| Tomsk oblast | Russia | 84.117 | 57.217 | Eastern | Abramson et al., 2009 |
| Lake Teletskoe | Russia | 87.683 | 51.617 | Eastern | Abramson et al., 2009 |

**REFERENCES**

Abramson, N. I., Rodchenkova, E. N., & Kostygov, A. Y. (2009). Genetic variation and phylogeography of the bank vole (*Clethrionomys glareolus*, Arvicolinae, Rodentia) in Russia with special reference to the introgression of the mtDNA of a closely related species, red-backed vole (*Cl. rutilus*). *Russian Journal of Genetics*, *45*(5), 533–545. https://doi.org/10.1134/S1022795409050044

Colangelo, P., Aloise, G., Franchini, P., Annesi, F., & Amori, & G. (2011). Mitochondrial DNA reveals hidden diversity and an ancestral lineage of the bank vole in the Italian peninsula. https://doi.org/10.1111/j.1469-7998.2011.00884.x

Deffontaine, V., Ledevin, R., Fontaine, M. C., Quéré, J.-P., Renaud, S., Libois, R., & Michaux, J. R. (2009). A relict bank vole lineage highlights the biogeographic history of the Pyrenean region in Europe. *Molecular Ecology*, *18*(11), 2489–2502. https://doi.org/10.1111/j.1365-294X.2009.04162.x

Deffontaine, V., Libois, R., Kotlík, P., Sommer, R., Nieberding, C., Paradis, E., … Michaux, J. R. (2005). Beyond the Mediterranean peninsulas: evidence of central European glacial refugia for a temperate forest mammal species, the bank vole (*Clethrionomys glareolus*). *Molecular Ecology*, *14*(6), 1727–1739. https://doi.org/10.1111/j.1365-294X.2005.02506.x

Kotlík, P., Deffontaine, V., Mascheretti, S., Zima, J., Michaux, J. R., & Searle, J. B. (2006). A northern glacial refugium for bank voles (*Clethrionomys glareolus*). *Proceedings of the National Academy of Sciences*, *103*(40), 14860–14864. https://doi.org/10.1073/pnas.0603237103

Kotlík, P., Marková, S., Vojtek, L., Stratil, A., Slechta, V., Hyršl, P., & Searle, J. B. (2014). Adaptive phylogeography: Functional divergence between haemoglobins derived from different glacial refugia in the bank vole. *Proceedings of the Royal Society B: Biological Sciences*, *281*(1786). https://doi.org/10.1098/rspb.2014.0021

Marková, S., Horníková, M., Lanier, H. C., Henttonen, H., Searle, J. B., Weider, L. J., & Kotlík, P. (2020). High genomic diversity in the bank vole at the northern apex of a range expansion: The role of multiple colonizations and end‐glacial refugia. *Molecular Ecology*, *29*(9), 1730–1744. https://doi.org/10.1111/mec.15427

Searle, J. B., Kotlík, P., Rambau, R. V., Marková, S., Herman, J. S., & McDevitt, A. D. (2009). The Celtic fringe of Britain: Insights from small mammal phylogeography. *Proceedings of the Royal Society B: Biological Sciences*, *276*(1677), 4287–4294. https://doi.org/10.1098/rspb.2009.1422

Strážnická, M., Marková, S., Searle, J., & Kotlík, P. (2018). Playing hide-and-seek in beta-globin genes: gene conversion transferring a beneficial mutation between differentially expressed gene duplicates. *Genes*, *9*(10), 492. https://doi.org/10.3390/genes9100492

Wójcik, J. M., Kawałko, A., Marková, S., Searle, J. B., & Kotlík, P. (2010). Phylogeographic signatures of northward post-glacial colonization from high-latitude refugia: a case study of bank voles using museum specimens. *Journal of Zoology*, *281*(4), 249–262. https://doi.org/10.1111/j.1469-7998.2010.00699.x
