# Supplementary material for: Niche differentiation in a postglacial colonizer, the bank vole Clethrionomys glareolus
Source: Ecol Evol. 2021 May 17;11(12):8054–70. doi: 10.1002/ece3.7637 (PMC8216960; doi:10.1002/ece3.7637)
Supplement: Supplementary file 2 — Appendix S2 [file ECE3-11-8054-s002.docx]

**Supporting Information**

**Niche differentiation in a postglacial colonizer, the bank vole *Clethrionomys glareolus***

Marco A. Escalante, Michaela Horníková, Silvia Marková and Petr Kotlík

**Appendix S2.** Supporting information.

**Table S2.1.** The ability of the model constructed for each lineage to predict the distribution of the other lineages assuming three different presence thresholds: minimum training presence (MTP), 5 percentile training presence (5 PTP) and 10 percentile training presence (10 PTP).

**Table S2.2.** Niche overlaps between models calculated for the bank vole (species-level) and each independent lineage using two different sets of predictor (climatic) variables (Set 1 and Set 2). Niche overlap, quantified by Schoener’s *D* and Hellinger’s *I*, is evaluated in geographical (*G*) as well as environmental (*E*) space.

**Fig. S2.1** Response curves showing how each environmental variable included in Set 1 and Set 2 affects the Maxent prediction of suitable habitat for the bank vole and each lineage. The x-axis of the variables represents their ranges for the study area, while the y-axis represents the predicted suitability of the focus variable: Mean Diurnal Range (Bio 2), Isothermality (Bio 3), Temperature Annual Range (Bio 7), Mean Temperature of Wettest Quarter (Bio 8), Mean Temperature of Driest Quarter (Bio 9), Mean Temperature of Warmest Quarter (Bio 10), Precipitation Seasonality (Bio 15), Precipitation of Wettest Quarter (Bio 16), Precipitation of Driest Quarter (Bio 17) and Precipitation of Warmest Quarter (BIO18).

**Fig.S2.2** Current habitat suitability for the bank vole and each of the four phylogeographic lineages predicted using two different sets of predictor (climatic) variables (Set 1 and Set 2) and applying three thresholds. The boundary of the bank vole distribution range (IUCN 2010) is represented by the white polygons.

**Fig. S2.3** Composite prediction from the models constructed for the individual bank vole lineages with Set 1 of climatic variables. The boundary of the bank vole distribution range (IUCN 2010) is represented by the white polygon.

**Fig. S2.4** Composite prediction from the models constructed for the individual bank vole lineages with Set 2 of climatic variables. The boundary of the bank vole distribution range (IUCN 2010) is represented by the white polygon.

**Fig. S2.5** Mid-Holocene niche projections for the bank vole and each lineage based on (a) CCSM4 and (b) MIROC-ESM paleoclimate models. The boundary of the bank vole distribution range (IUCN 2010) is represented by the white polygons.

**Fig. S2.6** Last Glacial Maximum niche projections for the bank vole and each lineage based on (a) CCSM4 and (b) MIROC-ESM paleoclimate models. The boundary of the bank vole distribution range (IUCN 2010) is represented by the white polygons.

**Fig. S2.7** Principal Component Analysis of Set 1 and Set 2 of predictor variables, showing the occupied niches (blue) against the background points from each model (orange) and a 100,000-point sample of the continuous multidimensional environmental space (black). The plots show that the different lineages occupy different, only partly overlapping subsets of the available environmental combinations, which are a subset of the continuous environmental space bounded by the minimum and maximum value of the respective climate rasters.

**Table S2.1.** The ability of the model constructed for each lineage to predict the distribution of the other lineages assuming three different presence thresholds: minimum training presence (MTP), 5 percentile training presence (5 PTP) and 10 percentile training presence (10 PTP).

| **Model** | **Lineage (N)** | **Predicted with Set 1** | | | **Predicted with Set 2** | | |
| --- | --- | --- | --- | --- | --- | --- | --- |
|  |  | **MTP** | **5 PTP** | **10 PTP** | **MTP** | **5 PTP** | **10 PTP** |
| Carpathian | Eastern (85) | 63 (74.1%) | 43 (50.6%) | 42 (49.4%) | 57 (67.1%) | 38 (44.7%) | 37 (43.5%) |
|  | Southern (36) | 28 (77.8%) | 15 (41.7 %) | 14 (38.9%) | 35 (97.2%) | 24 (66.7%) | 21 (58.3%) |
|  | Western (138) | 134 (97.1%) | 105 (76.1%) | 101 (73.2%) | 138 (100%) | 133 (96.4%) | 126 (91.3%) |
| Eastern | Carpathian (107) | 93 (86.9%) | 75 (70.1%) | 69 (64.5%) | 97 (90.7%) | 78 (72.3%) | 71 (63.4%) |
|  | Southern (36) | 9 (25%) | 3 (8.3 %) | 2 (5.6%) | 28 (77.8%) | 13 (36.1%) | 10 (27.8%) |
|  | Western (138) | 110 (79.7%) | 76 (55.1%) | 65 (47.1%) | 128 (92.8%) | 83 (60.1%) | 70 (50.7%) |
| Southern | Carpathian (107) | 35 (32.7%) | 20 (18.7%) | 18 (16.8%) | 86 (80.3%) | 28 (26.2%) | 20 (18.7%) |
|  | Eastern (85) | 16 (18.8%) | 11 (12.9%) | 5 (5.9%) | 36 (42.4%) | 11 (12.9%) | 7 (8.2%) |
|  | Western (138) | 86 (62.3%) | 53 (38.4%) | 30 (21.7%) | 121 (87.7%) | 74 (53.6%) | 55 (39.9%) |
| Western | Carpathian (107) | 99 (92.5%) | 81 (75.7%) | 57 (53.3%) | 92 (86%) | 70 (65.4%) | 63 (58.9%) |
|  | Eastern (85) | 44 (51.8%) | 26 (30.6%) | 16 (18.8%) | 33 (38.8%) | 21 (24.7%) | 17 (20%) |
|  | Southern (36) | 25 (69.4%) | 13 (36.1%) | 8 (22.2%) | 30 (83.3%) | 24 (66.7%) | 19 (52.8%) |

**Table S2.2.** Niche overlaps between models calculated for the bank vole (species-level) and each independent lineage using two different sets of predictor (climatic) variables (Set 1 and Set 2). Niche overlap, quantified by Schoener’s *D* and Hellinger’s *I*, is evaluated in geographical (*G*) as well as environmental (*E*) space.

|  |  |  |  | **Model A *versus* Model B** | | | | |
| --- | --- | --- | --- | --- | --- | --- | --- | --- |
|  |  |  |  | ***G*-space** | |  | ***E*-space** | |
| **Predictors** | **Model A** | **Model B** |  | ***D*** | ***I*** |  | ***D*** | ***I*** |
| Set 1 | Species-level | Carpathian |  | 0.58 | 0.84 |  | 0.33 | 0.60 |
|  |  | Eastern |  | 0.73 | 0.92 |  | 0.25 | 0.50 |
|  |  | Southern |  | 0.46 | 0.78 |  | 0.34 | 0.61 |
|  |  | Western |  | 0.51 | 0.79 |  | 0.25 | 0.50 |
| Set 2 | Species-level | Carpathian |  | 0.59 | 0.86 |  | 0.55 | 0.81 |
|  |  | Eastern |  | 0.76 | 0.94 |  | 0.33 | 0.61 |
|  |  | Southern |  | 0.47 | 0.79 |  | 0.37 | 0.58 |
|  |  | Western |  | 0.42 | 0.73 |  | 0.40 | 0.65 |

.
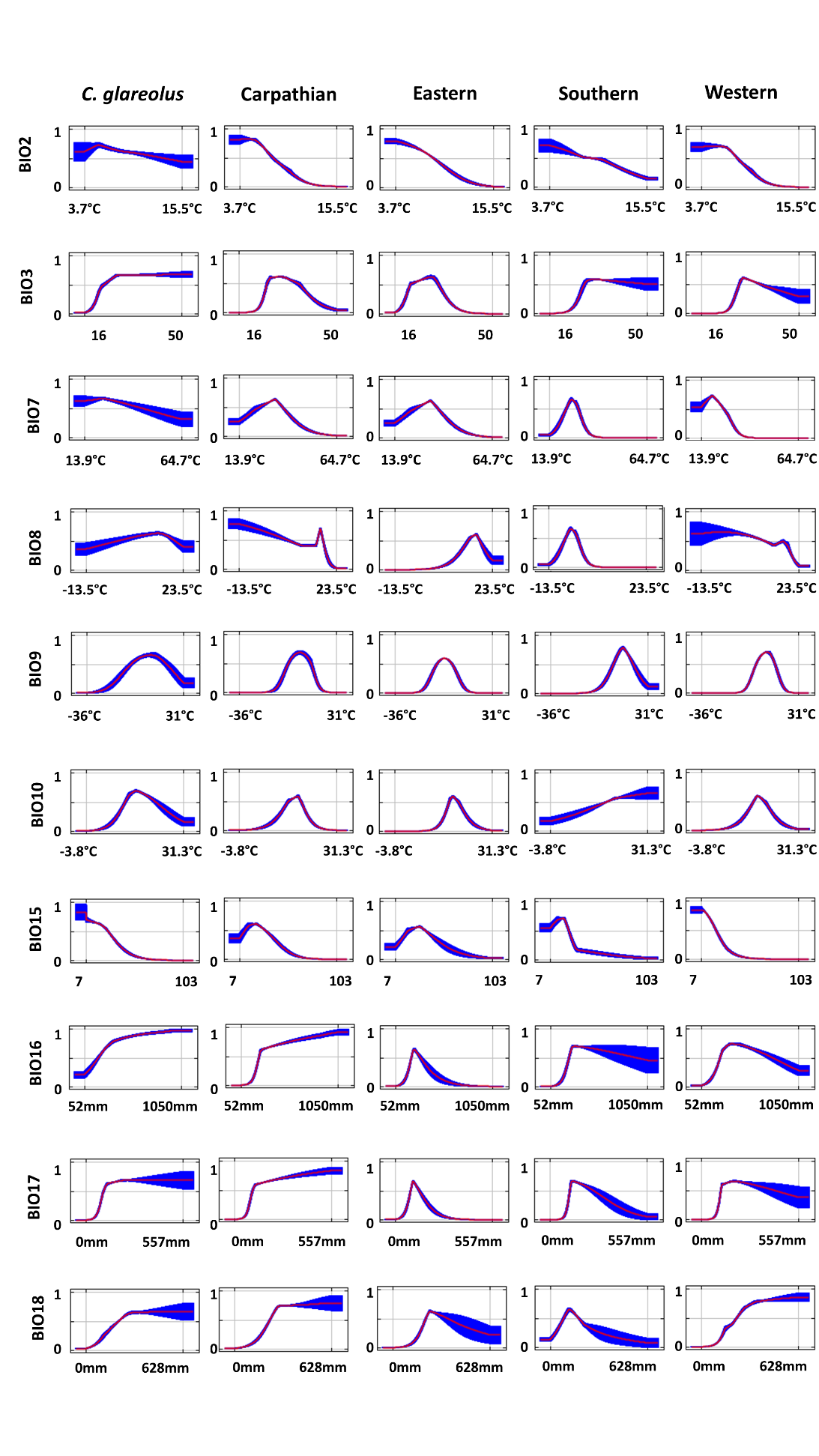


**Fig. S2.1** Response curves showing how each environmental variable included in Set 1 and Set 2 affects the Maxent prediction of suitable habitat for the bank vole and each lineage. The x-axis of the variables represents their ranges for the study area, while the y-axis represents the predicted suitability of the focus variable: Mean Diurnal Range (Bio 2), Isothermality (Bio 3), Temperature Annual Range (Bio 7), Mean Temperature of Wettest Quarter (Bio 8), Mean Temperature of Driest Quarter (Bio 9), Mean Temperature of Warmest Quarter (Bio 10), Precipitation Seasonality (Bio 15), Precipitation of Wettest Quarter (Bio 16), Precipitation of Driest Quarter (Bio 17) and Precipitation of Warmest Quarter (BIO18).


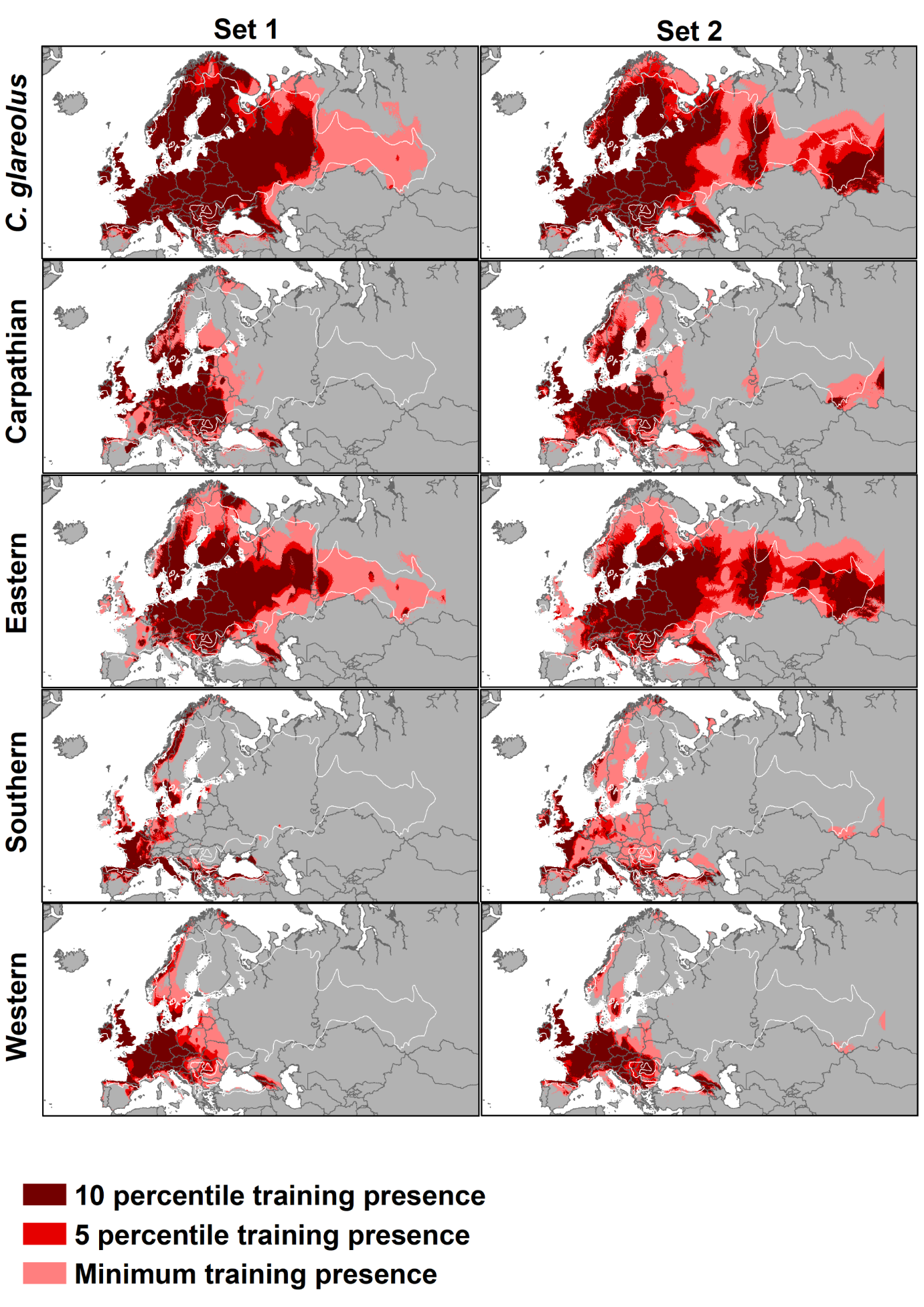


**Fig.S2.2** Current habitat suitability for the bank vole and each of the four phylogeographic lineages predicted using two different sets of predictor (climatic) variables (Set 1 and Set 2) and applying three thresholds. The boundary of the bank vole distribution range (IUCN 2010) is represented by the white polygons.


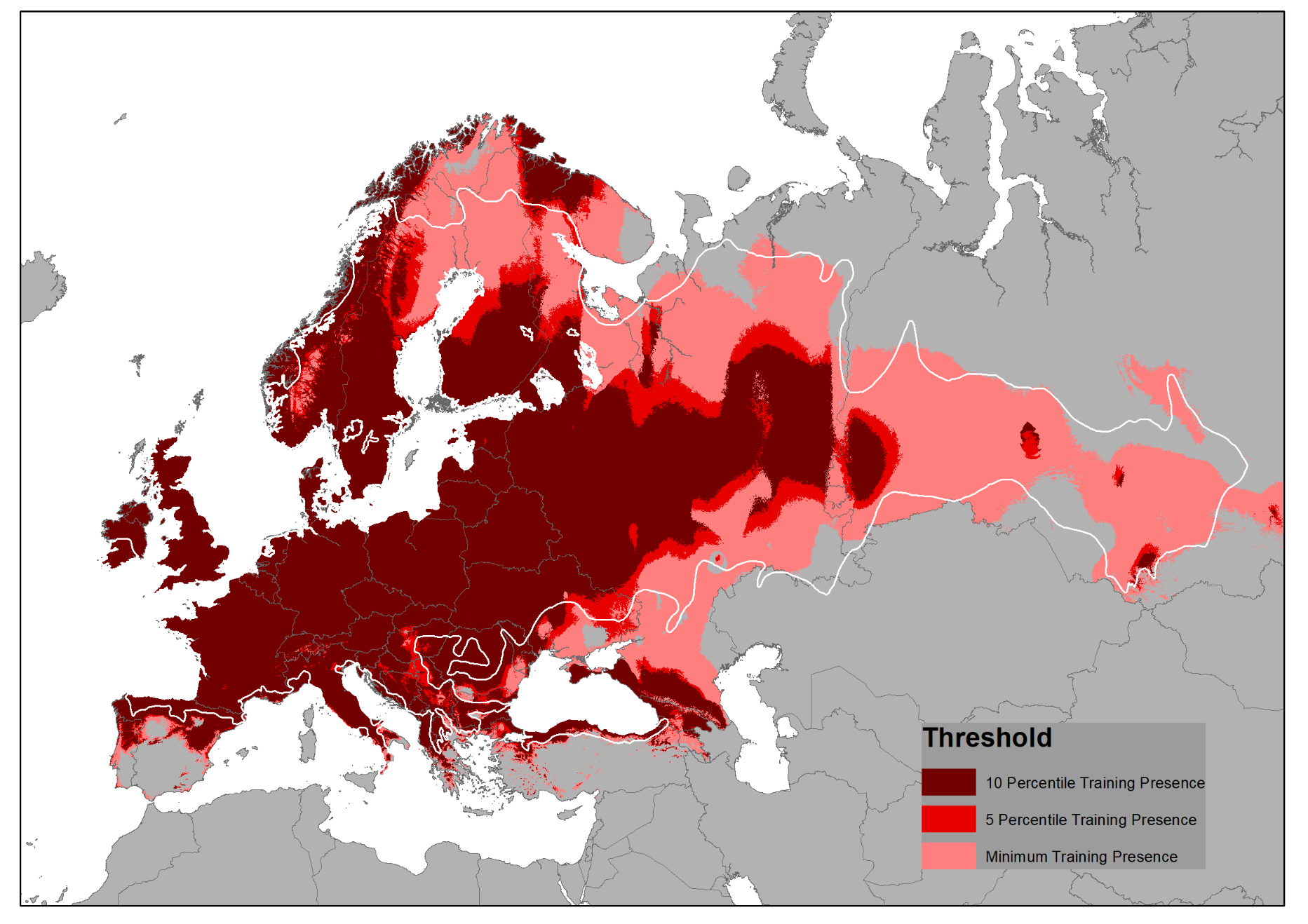


**Fig. S2.3** Composite prediction from the models constructed for the individual bank vole lineages with Set 1 of climatic variables. The boundary of the bank vole distribution range (IUCN 2010) is represented by the white polygon.


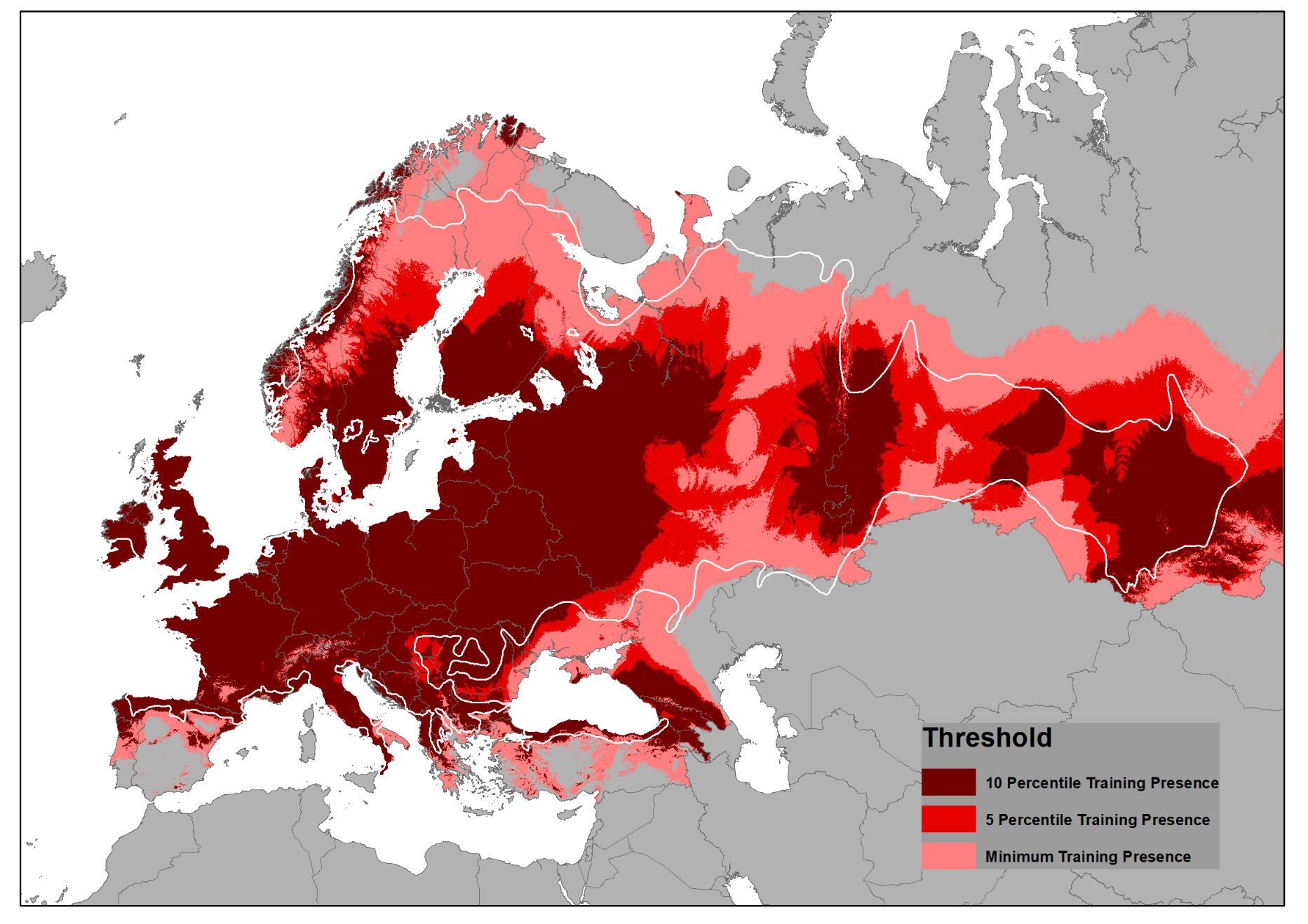


**Fig. S2.4** Composite prediction from the models constructed for the individual bank vole lineages with Set 2 of climatic variables. The boundary of the bank vole distribution range (IUCN 2010) is represented by the white polygon.

**
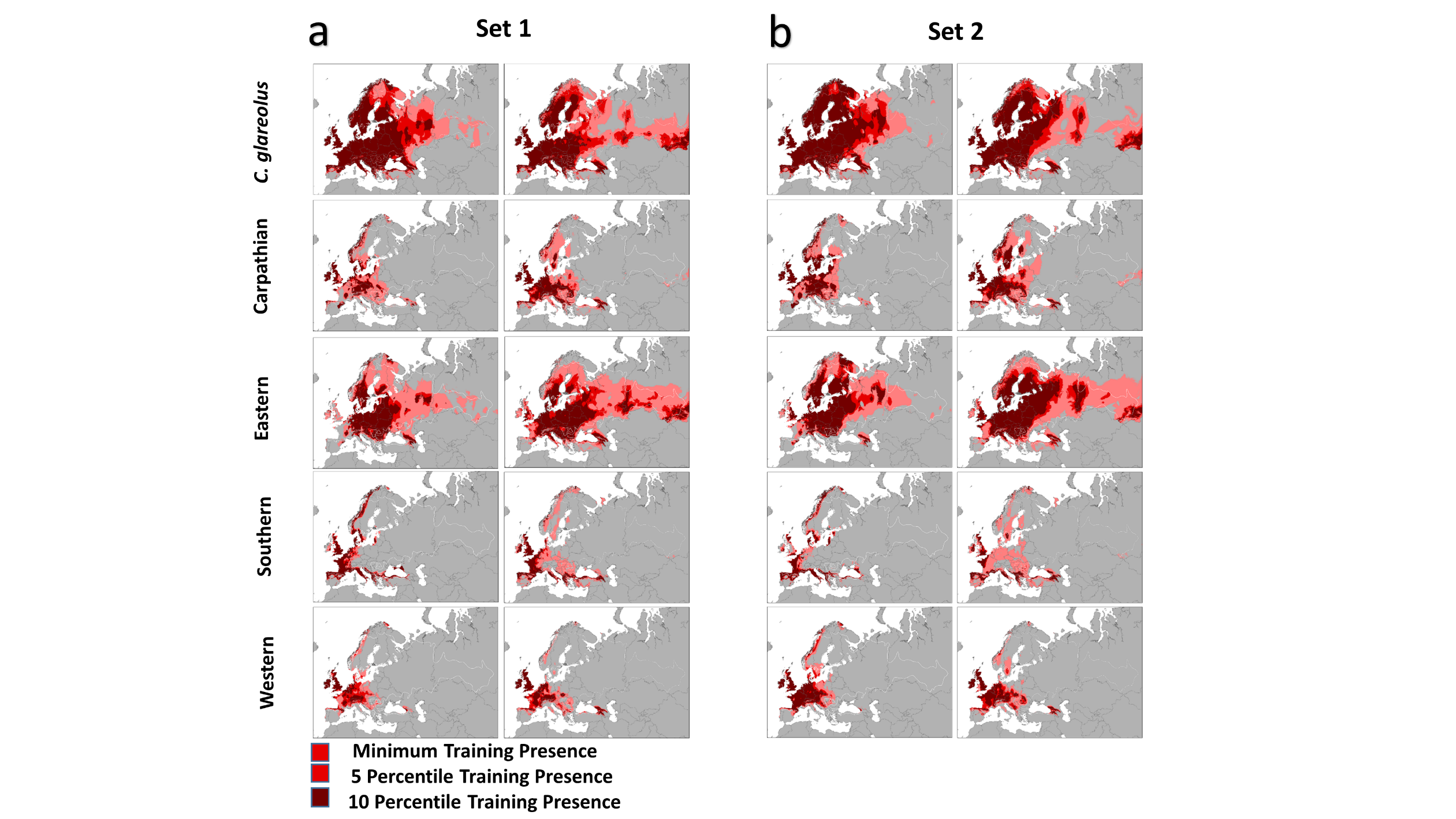
**

**Fig. S2.5** Mid-Holocene niche projections for the bank vole and each lineage based on (a) CCSM4 and (b) MIROC-ESM paleoclimate models. The boundary of the bank vole distribution range (IUCN 2010) is represented by the white polygons.


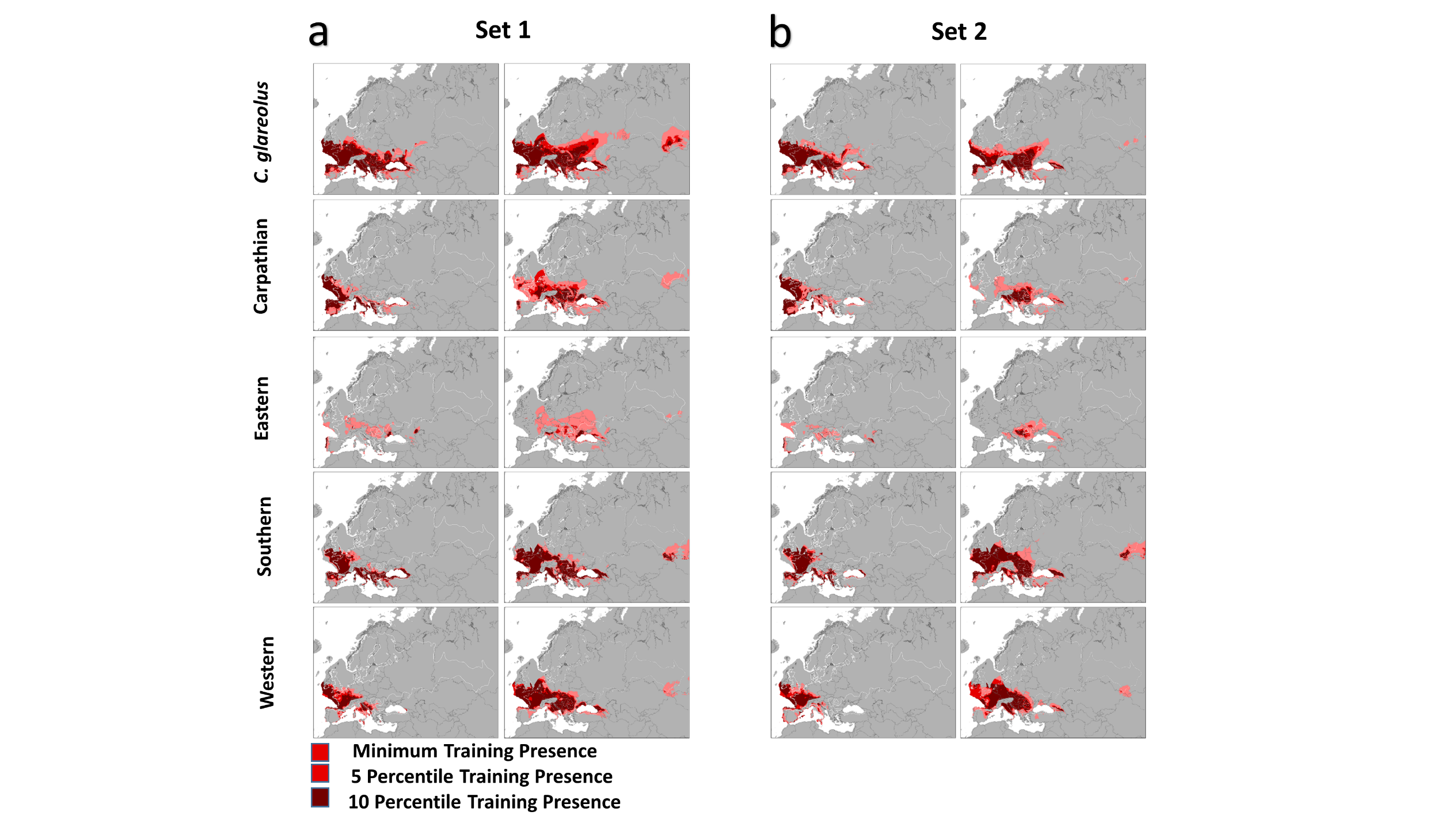


**Fig. S2.6** Last Glacial Maximum niche projections for the bank vole and each lineage based on (a) CCSM4 and (b) MIROC-ESM paleoclimate models. The boundary of the bank vole distribution range (IUCN 2010) is represented by the white polygons.


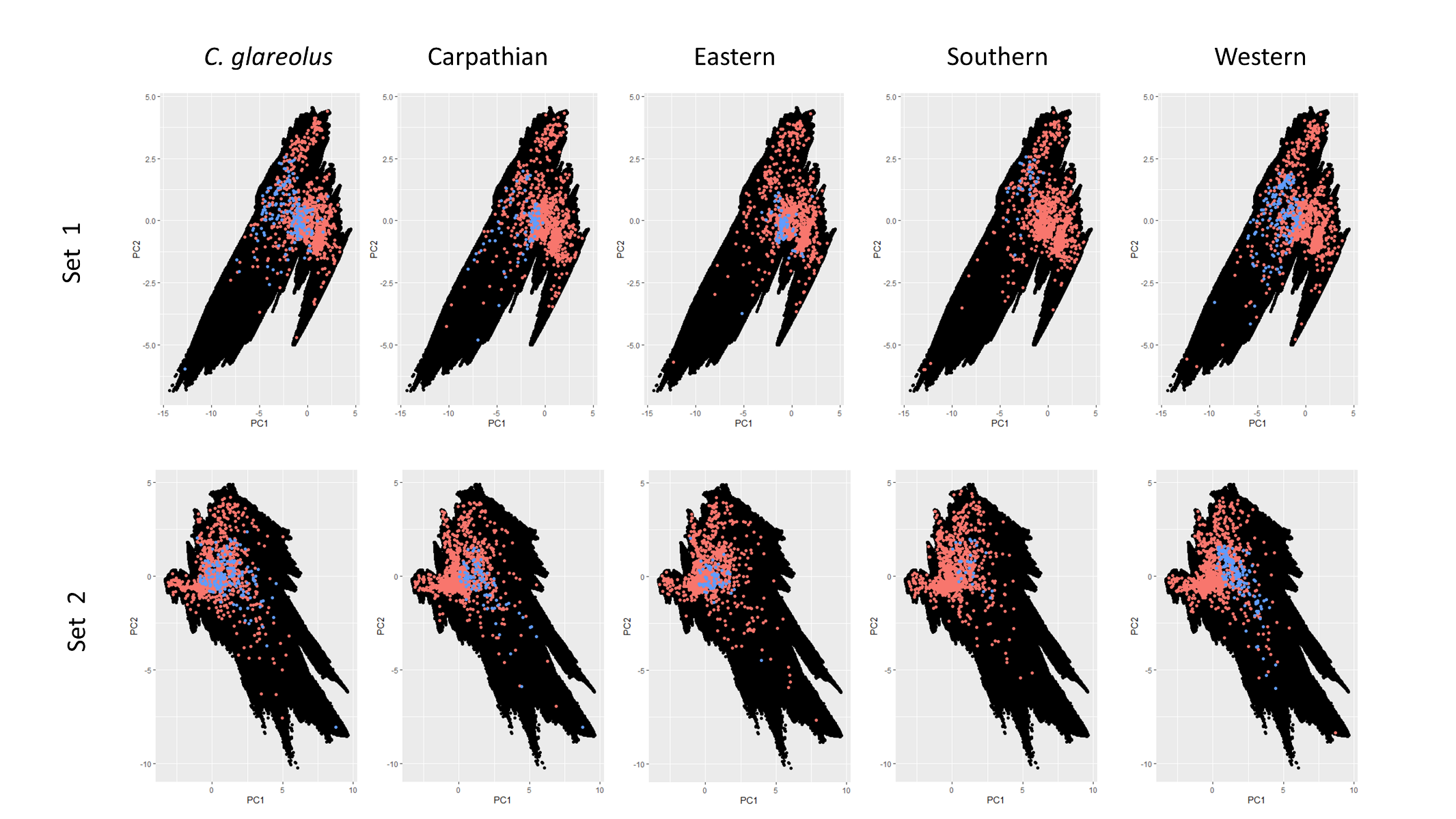


**Fig. S2.7** Principal Component Analysis of Set 1 and Set 2 of predictor variables, showing the occupied niches (blue) against the background points from each model (orange) and a 100,000-point sample of the continuous multidimensional environmental space (black). The plots show that the different lineages occupy different, only partly overlapping subsets of the available environmental combinations, which are a subset of the continuous environmental space bounded by the minimum and maximum value of the respective climate rasters.
